# Genera of the Protura of the World: diagnosis, distribution, and key

**DOI:** 10.3897/zookeys.772.24410

**Published:** 2018-07-06

**Authors:** Loris Galli, Julia Shrubovych, Yun Bu, Matteo Zinni

**Affiliations:** 1 Dipartimento di Scienze della Terra, dell’Ambiente e della Vita – Università di Genova, Corso Europa 26, 16132 Genova, Italy; 2 Institute of Systematics and Evolution of Animals, Polish Academy of Sciences, Sławkowska 17, Pl 31-016 Krakow, Poland; 3 State Museum of Natural History, Ukrainian Academy of Sciences, Teatral’na 18, UA 79008 Lviv, Ukraine; 4 Institute of Soil Biology, Biology Centre, Czech Academy of Sciences, Na Sádkách 7, 370 05 České Budějovice, Czech Republic; 5 Natural History Research Center, Shanghai Natural History Museum, Shanghai Science & Technology Museum, 399 Shanhaiguan Road, Shanghai, 200041, China

**Keywords:** distribution, key to genera, Protura, World

## Abstract

Protura are known all over the world with more than 800 described species belonging to three different orders (Acerentomata, Sinentomata, and Eosentomata) and seven families (Hesperentomidae, Protentomidae, Acerentomidae, Fujientomidae, Sinentomidae, Eosentomidae, and Antelientomidae). At present 76 genera are known worldwide. In this paper a description of the diagnostic characters of these genera and an updated key for their identification are reported.

## Introduction

Since their discovery (Silvestri 1907), the knowledge of Protura has rapidly increased all over the world thanks to the careful research of many specialists. For example, the number of worldwide genera cited by Tuxen (1964), Nosek (1978a), Yin (1983) and Szeptycki (2007) was 22, 40, 54 and 72, respectively. Four new genera (*Nanshanentulus* Bu & Yin, 2007, *Osientomon* Nakamura, 2010, *Liaoxientulus* Wu & Yin, 2011, *Nosekientomon* Shrubovych et al., 2014b) have been described after the publication of Szeptycki’s Catalogue (2007), but since Yin (1983) there has not been published an updated key for the worldwide genera of Protura. In the belief that such a key is an essential tool for those researchers who want to study in detail this still poorly known taxon, this paper describes the diagnostic characters of the 76 genera known to date worldwide, accompanied by a key for their identification.

The diagnostic characters used at a generic level for Protura have changed and increased in number thanks to the contributions from different authors during the last century. In the first papers published on Protura (Berlese 1908a, 1908b, 1909; Silvestri 1909) just four genera (*Acerentomon*, *Acerentulus*, *Proturentomon*, and Eosentomon – the possibility of subgenus Acerella was suggested in Berlese, 1909) were described mainly based on the presence/absence of a tracheal system, presence/absence of a rostrum, shape and size of mouthparts (maxillae, mandibles, maxillary, and labial palps), the number of segments of abdominal appendages and presence/absence of teeth on the lid (comb) covering the large glands on the sides of tergite VIII. In 1921 Ewing redescribed the genera *Eosentomon*, *Acerentomon*, and *Acerentulus* and described three new genera *Protentomon*, *Acerentuloides*, and *Microentomon* (the last one subsequently rejected). In his paper he stressed the importance of the size of abdominal appendages (one or two-segmented) and introduced for the diagnosis of genera the importance of tergal plates, the shape and size of tergal apodemes on the meso- and metanotum and on the first eight urotergites and the presence of transverse rows of dorsal setae on abdominal segments. The same characters, with reference also to the comb of urotergite VIII, were used by this author in his 1936 Synopsis of the genera of Protura. In a more extensive paper Ewing (1940) described genera of North American Protura based on dorsal apodemes, labrum, maxillae, mandibles, the number of transverse rows of setae on abdominal segments, presence of pectinated structures, characteristics and relative size of legs, colour and degree of sclerotization of cuticle (the size of abdominal appendages was elevated as a diagnostic character at a family level). Meanwhile, labial and tarsus ratios (LR and TR), chaetotaxy and details of both foretarsal sensilla/setae and female squama genitalis shape were introduced by Womersley, Ionescu and Condé, respectively, but such characters were used almost exclusively for new species description. In 1959, in their Synopsis of the Japanese Protura, Imadaté and Yosii described the new genus *Nipponentomon* based on previously used characters but included the number of setae shared by the four species of the genus on some of the abdominal segments and the distribution and shape of the abdominal pectinated structures. In 1964 Tuxen published his valuable monograph on the World Protura. Each genus was identified using characters such as abdominal appendages (size, number of setae and their relative size), shape and relative position of key sensilla on the foretarsus, presence and size of a rostrum, development of maxillary and labial palps, and shape of the maxillary glands. However, in addition to the important diagnostic features Tuxen provided meticulous descriptions of every part of the body morphology: e.g., the number of setae and sensilla on the labial and maxillary palps, the structure of all foretarsal sensilla present (and noting those absent), and the abdominal chaetotaxy. A similar scheme was followed by Nosek (1973) and Imadaté (1974) in their monographs on European and Japanese Protura, respectively, but the last author added the chaetotaxy of nota and, for Acerentomidae, the position of seta *P3* on abdominal tergites II–VI (diagnostic character he had suggested in 1964) in his diagnoses of genera. At the same time, Rusek (1974) examined in detail the taxonomy of Acerentomidae, redescribing older genera and introducing five new genera (*Vesiculentomon*, *Imadateiella*, *Filientomon*, *Verrucoentomon*, and *Nosekiella*), based on the following diagnostic characters: number and relative length of setae on abdominal appendages, shape of the maxillary glands, shape of sensillum *t1* and relative position of some sensilla on the foretarsus (mainly *a*’ respect to *t2*, *d* respect to *e* and *c*, *f* respect to *g*), number of anterior setae on the metanotum and toothed rows on the hind border of the last abdominal segments. Nosek (1978a) published a paper on the genera of Protura of the world. In the diagnosis of each genus he described the labial and maxillary palps, mandibles, maxillary glands, pseudoculi, some main foretarsal sensilla (e.g. *t1* and *t3*), abdominal appendages, chaetotaxy of sternite VIII, striate band (in Acerentomidae), comb VIII, and female squama genitalis; other characters (like the number of anterior setae on metanotum) are provided just in a few cases. Three years later Tuxen (1981) undertook a taxonomical revision of Acerentomidae based on the degree of development of the striate band of abdominal segment VIII, stressing the importance of the hook-shaped design, the number and relative size of setae of abdominal appendages (legs) and other characters (chaetotaxy of metanotum, shape of foretarsal sensillum *t1*, labial palps and maxillary glands). Based on these features he introduced the new genera *Kenyentulus* (splitting *Gracilentulus*) and *Amphientulus* (from *Berberentulus*). The next year Tuxen and Yin (1982) revised Protentomidae and introduced the new genus *Neocondeellum* (from *Condeellum*) based on shape of pseudoculi, maxillary palps, foretarsal sensilla, abdominal appendages, presence of a serrated line on abdominal segment VIII, comb VIII and chaetotaxy. Characters such as the presence/absence of a tracheal system, thoracic and abdominal chaetotaxy, pseudoculi, maxillary glands, labial palps and shape of foretarsal sensilla were used by Yin (1983) to arrange her phylogeny of Protura, grouping the 54 then known genera into 17 subfamilies and eight families. Zhang and Yin (1984) revised the subfamily Anisentominae and created the new genera *Neanisentomon*, *Pseudanisentomon* and *Paranisentomon* based mainly on the presence/absence and shape of few foretarsal sensilla (*t2*, *f1*, *b’2*) and on the chaetotaxy of abdominal segment VIII. Szeptycki (1988) introduced the term porotaxy to define the distribution of pores on the body of proturans and suggested the possibility of using this character for the identification of genera. Since then porotaxy has been generally used in the description of new species. Szeptycki (1995a) summarized the knowledge about porotaxy of Protura and reiterated the taxonomic importance it could have at the level of genus and family. Szeptycki also (see 1997) introduced the analysis of the shape of accessory setae (especially of meso- and metanotum) as a taxonomically important character (at least for Acerentomata). Unfortunately, porotaxy and the shape of accessory setae of the majority of species are still unknown and, for many genera, their use as diagnostic characters is not practicable. Nevertheless, they are among those characters used by Shrubovych (2014) in her cladistic approach to the Acerentomidae of Northeastern Palearctic. Other useful diagnostic characters are the head chaetotaxy and porotaxy (Rusek et al. 2012); currently they are mainly provided in new species description, but some, like the presence/absence of postpseudocular seta, have been used for the diagnosis of new genera (e.g. Wu and Yin 2011).

## Diagnosis

Nosek’s (1978a) diagnostic framework is partly used and integrated with the new genera described and updated in accordance with the more recent taxonomic revisions and redescriptions of Nakamura (2010), Shrubovych and Rusek (2010), Shrubovych et al. (2012a), Shrubovych and Smykla (2012), Shrubovych et al. (2014b).

For the whole bibliographic references to genera description and distribution, we refer to the World Catalogue by Szeptycki (2007). Genera are listed according to Szeptycki’s system (2007).

The diagnostic characters available for each genus are given, following the same general scheme: characters related to the head (especially the labial palps, maxillary glands and pseudoculi), foretarsal sensilla and setae (presence/absence, position, size and shape), notal chaetotaxy, abdominal appendages (number of segments and setae), other abdominal characters (chaetotaxy, striate band, comb VIII) and shape of female squama genitalis. When available and useful for taxonomic diagnosis, other characters considered are the presence of a rostrum and the cephalic chaetotaxy. Those unifying characters reported in the order, family or subfamily diagnosis are not repeated in that of genera. For the characters described in the diagnosis and used in the key, refer to Tuxen (1964), Nosek (1973), Tuxen and Imadaté (1974), Tuxen (1981), Tuxen and Yin (1982), Yin (1999), Rusek et al. (2012) and Shrubovych (2014). For the appearance/shape of some characters (e.g. the foretarsal sensilla and the maxillary glands) would be appropriate to consult directely the type species description or those papers indicated in the remarks of the genera.

### Abbreviations

abd. abdominal segment/s,

abd. app. abdominal appendages,

Tg. abdominal tergite/s,

St. abdominal sternite/s,

*A*-setae anterior setae,

*P*-setae posterior setae,

*Pc* posterior central seta.

### Order ACERENTOMATA Yin, 1996

Tracheal system missing. Dilated canal of maxillary glands. Foretarsus typically with three dorsal sensilla (*t1*–*t3*), seven external (*a*–*g*) and three internal (*a*’–*c*’). Abd. app. with no more than four setae. Presence of pectinated structures on the abdomen. Glands on abd. VIII covered by combs. Abd. VIII with striate band (fully developed or reduced). Female squama genitalis without processus sternalis but with acrostylus.

#### Family Hesperentomidae Price, 1960

Labial palps well developed, with tuft of setae. Maxillary glands long and dilated. Abd. app. I two-segmented, with terminal vesicle and four setae; II–III two-segmented or uni-segmented. Striate band without distinct striae. Comb VIII rectangular with small teeth. Female squama genitalis with pointed acrostyli.

#### Subfamily Hesperentominae Price, 1960

Maxillary palps with tuft of setae and two sensilla. Labial palps with tuft of setae and three distinct setae. Canal of maxillary glands with a long dilation. Pseudoculi open proximally. All foretarsal sensilla present, with addition of *c*”. Metanotum with median setae and two pairs of *A*-setae. Abd. app. I and II two-segmented, each with terminal vesicle and four setae; III two-segmented or uni-segmented with two setae.

##### 1 – *Hesperentomon* Price, 1960

Labial palps without basal sensillum. Canal of maxillary glands dilated as a long parallel-sided sac for most of its length, narrowing proximally. Pseudoculi pear-like, strongly elevated and with a median S-shaped cleft. Foretarsal sensilla setiform or longish willow leaf-shaped. *S* seta long, straight and pointed. Abd. app. I–III two-segmented, each with four setae. St. II–VI with *Pc* seta (absent only on St. IV–VI in few species). St. VIII with a single posterior row of six setae. Abd. VIII with a serrated line at the anterior border. Fine teeth at the hind border of St. IX–X.

Type species. *Hesperentomon macswaini* Price 1960.

Distribution. East and Central Asia, North America.

Remarks. For figures and a key to species of genus *Hesperentomon*, see Shrubovych (2010).

##### 2 – *Ionescuellum* Tuxen, 1960

Labial palps with basal sensillum. Canal of maxillary glands with calyx, a long dilation and a second round dilation distal to calyx. Pseudoculi oval and open proximally. Abd. app. I-II two-segmented, each with terminal vesicle and four setae; appendage III uni-segmented with two setae. St. II–VI without *Pc* seta. St. VIII with 4/0 setae.

Type species. *Paraentomon carpaticum* Ionesco, 1930.

Distribution. Europe.

Remarks. Figures and a key to species of genus *Ionescuellum* are available in Rusek and Stumpp (1989).

#### Subfamily Huhentominae Yin, 1983

Labial palps well developed with tuft of setae and three distinct setae, without basal sensillum. Metanotum with one or two pairs of *A*-setae. Abd. app. I two-segmented; II–III uni-segmented, each with three setae, the median apical one approx. twice the length of the others. St. IV–VII each with *Pc* seta; St. VIII with 2/4 setae.

##### 3 – *Huhentomon* Yin, 1977

Canal of maxillary glands extremely long; middle part approx. 3.5 times of hind part, with a tiny racemose appendix distally. Pseudoculi with a median S-shaped opening. Foretarsal sensilla of *Hesperentomon* type; sensillum *t1* filiform; *t3* rather long, sensilliform; *b*’, *c*’ and *c*” distal, close to *t3*; *d* nearly halfway between *c* and *e*, distal to *t2*.

Type species. *Huhentomon plicatunguis* Yin, 1977.

Distribution. China, Japan.

Remarks. Figures and comparison between the two species of genus *Huhentomon* are available in Imadaté (1989).

##### Family Protentomidae Ewing, 1936

Small bodied Protura. Maxillary glands with smooth globular calyx. Foretarsal sensillum *t1* sometimes missing (in all the species belonging to genus *Protentomon* and some of those belonging to *Proturentomon*). Metanotum with one or two pairs of *A*-setae. Abd. app. I–II two-segmented, each with terminal vesicle and three or four setae; III two-segmented or uni-segmented with two or three setae.

#### Subfamily Hinomotentominae Yin, 1999

Abd. app. two-segmented, each with terminal vesicle and four setae. Female squama genitalis with pointed acrostyli.

##### 4 – *Hinomotentomon* Imadaté, 1973

Maxillary palps with tuft of setae and two sensilla. Labial palps with tuft of setae, three distinct setae and basal sensillum. Canal of maxillary glands with heart-shaped calyx with verrucose appendices and a proximal dilation. Pseudoculi with a large triangular prolongation in the proximal part. Foretarsal sensillum *a*’ short; *b*’ and *c*’ missing. Metanotum with two pairs of *A*-setae. St. IV–V without *Pc* seta. St. VIII with four setae. Comb VIII with 10 small teeth.

Type species. *Hesperentomon nipponicum* Imadaté, 1964.

Distribution. Japan.

Remarks. For figures, see Imadaté (1973).

#### Subfamily Condeellinae Tuxen & Yin, 1982

Labial palps with tuft of setae. Metanotum with median setae and two pairs of *A*-setae. Abd. app. I–II two-segmented, each with four setae; III uni-segmented with two or three setae. Female squama genitalis with pointed acrostyli.

##### 5 – *Condeellum* Tuxen, 1963

Labial palps without basal sensillum. Sensillum of maxillary palps slender and pointed. Canal of maxillary glands with heart-shaped dilation, the proximal part only dilated along half its length; no proximal globule. Pseudoculi fairly large (PR = 8–10), circular, elevated over the head surface, without lever. Four dorsal setae between pseudoculi (*d4* and *sd4*) and two setae lateral to these (*l3*). Foretarsi with only four setiform sensilla in the exterior and interior side, respectively: *a*, *b*, *f* and *a*’; sensilla *t1* and *t2* are long, thin and pointed. Seta *S* long. Abd. app. III uni-segmented with three setae, the median apical one approx. twice the length of the others. St. I–VII with four *A*-setae; IV–VI with *Pc* seta. St. VIII with six setae in a row. Comb VIII mostly without teeth.

Type species. *Proturentomon regale* Condé, 1958.

Distribution. Subtropical China, Pacific Islands, Tropical Asia, Reunion.

Remarks. Figures and comparison between species of genus *Condeellum* are available in Tuxen and Yin (1982) and Imadaté (1991).

##### 6 – *Neocondeellum* Tuxen & Yin, 1982

Two short and blunt sensilla on maxillary palps. Labial palps without basal sensillum. Canal of maxillary glands, with a globular bladder, in connection with the canal, but not with a simple calyx. Pseudoculi small (PR = 14–18), with a short lever. Four dorsal setae between the pseudoculi (*d4* and *sd4*); lateral setae *l3* missing. Foretarsi with only four sensilla in the exterior and interior side, respectively: *a*, *b*, *f* and *a*’. Abd. app. III uni-segmented with three setae, the median apical one longer than the lateral. Anterior row of Tg. II–VII with at most six setae. Tg. VIII with a single serrate line (striate band) rarely with indication of an anterior border. St. IV–V without *Pc* seta. St. VIII with six setae. Comb VIII with approx. ten very small teeth.

Type species. *Condeellum brachytarsum* Yin, 1977.

Distribution. China, Japan, North America.

Remarks. For figures and comparison between species of genus *Neocondeellum*, see Tuxen and Yin (1982). For a more recent key to eight *Neocondeellum* spp., see Nakamura (1990).

##### 7 – *Paracondeellum* Yin, Xie & Zhang, 1994

Calyx of maxillary glands globular and smooth. Pseudoculi circular without lever. Foretarsal sensilla of the exterior side fully developed; *b*’ and *c*’ missing. Abd. app. III uni-segmented with two setae, the median apical longer than the lateral one. Tg. I with one pair of anterior setae; Tg. II–VII without *A*-setae. St. II–III with three *P*-setae; St. IV–V with nine *P*-setae; St. VIII with 4/0 setae.

Type species. *Condeellum dukouensis* Tang & Yin, 1988.

Distribution. China.

Remarks. For figures, see in Yin et al. (1994).

#### Subfamily Protentominae Ewing, 1936

Labial palps with tuft of setae. Pseudoculi with a long proximal prolongation. Foretarsi with foliaceous or bacilliform (long, parallel-sided, with blunt apex) sensilla. Metanotum with median setae and one or two pairs of *A*-setae. Abd. app. I–II two-segmented and III uni-segmented, with 4-4-2 or 3-3-2 setae. St. VIII with 4/0 setae. Female squama genitalis with knob-like acrostyli and two median papillae between styli.

##### 8 – *Protentomon* Ewing, 1921

Labial palps with or without basal sensillum. Canal of maxillary glands with disc-shaped dilation and sac-like proximal dilation. Pseudoculi mostly elliptical, with a narrow and long (more than pseudoculi) proximal prolongation. Foretarsi relatively short; *t1* absent; *c* sometimes missing. Metanotum with two pairs of *A*-setae (*A2*, *A4*). Comb VIII mostly without teeth, sometimes with very fine teeth, similarly to the hind margin of Tg. XII.

Type species. *Protentomon transitans* Ewing, 1921.

Distribution. Cosmopolitan.

Remarks. For figures, see Nosek (1973, 1976), Bernard (1975), Tuxen (1977a).

##### 9 – *Proturentomon* Silvestri, 1909

Labial palps with or without basal sensillum. Canal of maxillary glands with disc-shaped dilation and sac-like proximal dilation, with sharp proximo-lateral corner, terminating in doubled globule. Pseudoculi with a large triangular proximal prolongation of the same length of pseudoculi and of almost the same width distally. Foretarsi sometimes missing sensillum *t1*; *c* always present; sensilla on exterior and interior side of foretarsi are foliaceous or bacilliform, respectively. Seta *S* blunt, relatively short. Metanotum with one pair of *A*-setae (*A2*). Comb VIII with distinct teeth. The hind border of Tg. IX–XI serrated.

Type species. *Acerentomon minimum* Berlese, 1908.

Distribution. Holarctic.

Remarks. For figures, see Rusek (1975 – with a key to nine *Proturentomon* spp.), Szeptycki (1988) and Yin (1999).

#### Family Acerentomidae Silvestri, 1907

Mesonotum with a pair of median setae and two or three pairs of *A*-setae; metanotum with a pair of median setae and two to four pairs of *A*-setae. Abd. app. I two-segmented with four setae; II and III each uni-segmented with one to three setae. Abd. VIII with striate band.

##### Subfamily Berberentulinae Yin, 1983

Labial palps generally reduced (without tuft of setae) with two to four setae and one sensillum. Maxillary glands with generally smooth and often heart-shaped calyx. Foretarsal sensillum *t2* usually setiform/filiform. Meso- and metanotum each with two pairs of *A*-setae (*A2*, *A4*). Striate band on abd. VIII often reduced.

###### 10 – *Acerentuloides* Ewing, 1921

Maxillary palps with two spindle-like sensilla. Labial palps with a terminal tuft of setae and a sausage-shaped basal sensillum. Canal of maxillary glands with proximal part as long as the proximal part of fulcrum, ending with a row of small globules which increase in size proximally and end with two globules placed beside each other. Pseudoculi small. All foretarsal sensilla present, of average length; sensillum *t1* claviform; *t3* long lancet-like or finger-like; *d* closer to *c* than to *e*; *a*’ close to the base of *t1*. Abd. app. II–III each with three setae, the median apical one reduced to little more than its pit. Tg. II–VI with seta *P3* in anterior position. St. I–VII with three setae in anterior row. St. VIII with 4/2 setae. Striate band well developed. Comb VIII with a row of approx. ten equal small teeth. Some setae modified as short, thickened sensilla (*sd5* on head; *β1* and *δ4* on foretarsi; setae *P4* on metanotum, *M2* on prosternum and *A2* on thoracic sterna; accessory setae on Tg. and St. I–VI). Female squama genitalis tripartite with pointed acrostyli as in *Acerentulus*.

Type species. *Acerentuloides bicolor* Ewing, 1921.

Distribution. North America.

Remarks. For figures and diagnosis, see Shrubovych et al. (2017).

###### 11 – *Acerentulus* Berlese, 1908

Maxillary and labial palps with two and one sausage-shaped sensilla, respectively, and with a tuft of setae. Canal of maxillary glands with simple dilation, calyx sometimes weakly verrucose, proximal part rather long. Pseudoculi oval or circular with divided lid and short fork-shaped prolongation on the posterior margin of the lid. On foretarsi sensillum *t1* claviform; *t3* willow leaf-shaped; *a*’ usually broad; *b*’ always present. Abd. app. II–III each with three setae; the subapical is long, the lateral apical shorter and the median apical the shortest. Seta *P3* on Tg. II–VI placed anteriorly to the row of the other *P*-setae. St. I–VII with three anterior setae. St. VIII with 4/2 setae. Striate band on abd. VIII well developed. Comb VIII with approx. ten teeth. Female squama genitalis distally tripartite.

Type species. *Acerentomon confine* Berlese, 1908.

Distribution. Cosmopolitan.

Remarks. Figures and recent keys to the species of the *confinis* and *cunhai* group are available in Shrubovych et al. (2012b) and Shrubovych et al. (2014d), respectively.

###### 12 – *Amazonentulus* Yin, 1989

Labial palps reduced to three setae and a sensillum. Canal of maxillary glands of middle length, proximally bipartite. Foretarsal sensillum *t1* claviform; *t3* very long and awl-shaped; *b*’ absent. Abd. app. II–III each with two setae, the apical one shorter than half length the subapical. Tg. I–VII with uneven number of anterior setae. St. VIII with 4/0 setae. Striate band on abd. VIII reduced, without or with very short striae. Comb VIII with five to eight small teeth. Female squama genitalis with sharp pointed acrostyli.

Type species. *Berberentulus brasilianus* Nosek, 1973.

Distribution. South America.

Remarks. For figures, see Tuxen (1976).

###### 13 – *Amphientulus* Tuxen, 1981

Labial palps reduced to three setae and a sensillum. Canal of maxillary glands simple. Foretarsal sensillum *t1* claviform; *t3* jar-shaped; *b*’ present; *b* most often shorter than *c.* Abd. app. II–III each with a long subapical seta and a strong lateral apical one. St. VIII with 4/0 or 4/2 setae. Striate band on abd. VIII reduced (often showing an hook-shaped design). Comb VIII oblique, with large teeth. Female squama genitalis with long acrostyli.

Type species. *Berberentulus validus* Tuxen, 1967.

Distribution. Australia, New Zealand, South America, China, Madagascar (doubtful).

Remarks. For figures, see Tuxen (1967, 1986), Imadaté (1973), Nosek (1978b).

###### 14 – *Andinentulus* Tuxen, 1984

Labial palps reduced to three setae and a sausage-like sensillum. Proximal part of canal of maxillary glands almost as long as proximal branch of fulcrum. Foretarsi with all sensilla present; *t1* claviform; *t3* knob-shaped; *a*’ extremely long, situated between the insertions of *t1* and *t2*; *b*’ present; *d* near the base of *c.* Abd. app. II–III each with three setae. Tg. II–VII with seta *P3* in anterior position. Tg. II–V with six *A*-setae. St. I–VII with three *A*-setae. St. VIII with 4/2 setae. Striate band on abd. VIII reduced. Female squama genitalis with short basal arms, slender acrostyli with long and pointed apex.

Type species. *Andinentulus rapoporti* (Condé, 1963) (*Andinentulus ebbei* Tuxen, 1984 is synonymised after Shrubovych et al. 2014e).

Distribution. South America.

Remarks. For figures, see Shrubovych et al. (2014e).

###### 15 – *Australentulus* Tuxen, 1967

Labial palps reduced to three setae and a sensillum. Canal of maxillary glands with heart-shaped calyx and a proximal tripartite dilation. Foretarsal sensillum *t3* shaped as a bud rounded at apex, parallel-sided two to five times as long as broad; *t1* claviform; *a*’ broad; *b*’ present. Abd. app. II–III each with three setae. Seta *P3* on Tg. II–VI anterior to other *P*-setae row. St. VIII with 4/0 setae. Striate band well developed. Comb VIII with toothed margin. Female squama genitalis with pointed acrostyli.

Type species. *Acerentulus australiensis* Womersley, 1932.

Distribution. Australia, New Zealand, Tropical Asia, Madagascar.

Remarks. For a key to the identification of *Australentulus* species, see François (1994).

###### 16 – *Baculentulus* Tuxen, 1977

Labial palps reduced to two or three setae and a sensillum. Canal of maxillary glands simple, without appendages, with heart-shaped calyx. Foretarsal sensillum *t1* baculiform; *t3* jar-shaped; *b*’ present or absent. Abd. app. II–III each with two setae, the median apical very short, less than half the length of the subapical. Seta *P3* on Tg. II–VI anterior to other *P*-setae row. St. VIII with 4/0 setae. Abd. VIII with reduced striate band. Comb VIII more or less oblique. Female squama genitalis with pointed acrostyli.

Type species. *Berberentulus becki* Tuxen, 1976.

Distribution. Cosmopolitan.

Remarks. A recent key to the species of genus *Baculentulus* with foretarsal sensillum *b*’ is available in Bai & Bu (2013).

###### 17 – *Berberentulus* Tuxen, 1963

Labial palps reduced to two or three setae and a willow leaf-shaped or sausage-like sensillum. Maxillary palps with two sensilla. Canal of maxillary glands simple, with heart-shaped calyx and long or short proximal part, rarely with excrescences. Pseudoculi circular or oval. Foretarsal sensillum *t1* claviform; *t3* shaped as a willow leaf, or as a small jar or, sometimes, as a rounded knob; *d* very close to *c*; *b*’ usually missing. Empodium short. Abd. app. II–III each with two setae, the median apical less than 1/3^rd^ the length of the subapical. Seta *P3* on Tg. II–VI placed anteriorly to the row of the other *P*-setae. St. I–VII with three *A*-setae; St. VIII with 4/0 setae. Abd. VIII with reduced striate band formed by two rather close transverse lines, the more distal serrated, the proximal one more or less undulated with small teeth pointing backwards. Subcuticular parallel tubes are visible in correspondence to the missing striae. Comb VIII reduced with very small dispersed teeth. Female squama genitalis with long pointed acrostyli and broad styli.

Type species. *Acerentulus berberus* Condé, 1948.

Distribution. Cosmopolitan.

Remarks. For figures and keys to the species of genus *Berberentulus*, see Tuxen (1977b), Chao and Chen (1999).

###### 18 – *Bolivaridia* Bonet, 1942

Labial palps reduced to two setae and a sensillum. Maxillary palps with slender willow leaf-shaped sensilla. Canal of maxillary glands with heart-shaped calyx; the short proximal part with distinct lateral protuberance and with an ending “head of femur”-shaped dilation. Pseudoculi longitudinally divided, somewhat broader than long. Foretarsal sensillum *t1* claviform; *a*’ sword-shaped; *a* and *e* long and broad, spindle-shaped. Abd. app. II–III each with a single subapical seta. Tg. II–VI with *Ac* seta. St. I–VII with three *A*-setae; St. VIII with 4/0 setae. Striate band on abd. VIII reduced, with only short, blind striae extending from the posterior margin. Comb VIII concave, with small teeth. Female squama genitalis with long, broad and pointed acrostyli; basal apodeme short.

Type species. *Bolivaridia perissochaeta* Bonet, 1942.

Distribution. Pantropical.

Remarks. For a recent key to *Bolivaridia* species, see Bu and Palacios-Vargas (2012).

###### 19 – *Brasilentulus* Nosek, 1973

Labrum slightly prolonged into a rostrum. Labial palps reduced to three setae and a sensillum. Maxillary palps with two setiform sensilla. Canal of maxillary glands with heart-shaped calyx and long proximal part. All foretarsal sensilla present; long spindle-shaped sensillum *t3*; *t1* claviform (in *B. huetheri* Nosek the distal half bent forwards in a rather sharp angle); *b* placed close to *c.* Abd. app. II–III each with a long subapical seta and a short apical one (1/3^rd^ of the subapical). St. I–VII with three *A*-setae. St. VIII with 4/0 setae. Striate band complete. Comb VIII with teeth. Female squama genitalis with slender and pointed acrostyli.

Type species. *Brasilentulus huetheri* Nosek, 1973.

Distribution. Tropical America and Africa.

Remarks. Figures and a key to the species are available in Tuxen (1979).

###### 20 – *Brasilidia* Nosek, 1973

Labial palps reduced to three setae and a sensillum. Maxillary palps with two setiform sensilla. Canal of maxillary glands with heart-shaped calyx and relatively short proximal part, proximally tripartite. Pseudoculi longitudinally divided with short posterior fork-shaped prolongation. On the head seta *l3* is absent; seta *d6* (formerly “additional”) present. Foretarsal sensillum *d* situated nearer to *c* than to *e* (but not very close to *c*); *b*’ present; *t1* distinctly clavate (nearly “baculiform”); *t3* relatively long, cylindrical; setae *β1* and *δ4* not differentiated (of same shape as *δ1* – *δ3*). Seta *P2a* on meso- and metanotum nearer to *P3* than to *P2*; *P4a* on metanotum not modified (of “normal” shape). Abd. app. II–III each with three setae. Seta *P3* on Tg. II–V anterior to other *P*-setae row. St. VIII with 4/0 or 4/2 setae. Modified setae (meaning accessory setae on nota, Tg. and St. I–VII, and modified setae on head and on thoracic sterna) uniform over the whole body, as small, hair-like microchaetae. Striate band on abd. VIII reduced (with no distinct striae but just signs of them). Comb VIII with fine teeth. Female squama genitalis tripartite with pointed acrostyli.

Type species. *Brasilidia tropica* Nosek, 1973.

Distribution. South America.

Remarks. Figures and a recent key to the species are provided by Szeptycki and Bedano (2003).

###### 21 – *Chosonentulus* Imadaté & Szeptycki, 1976

Labial palps reduced to three setae and a broad sensillum. Canal of maxillary glands with racemose appendices, its proximal part rather short. Foretarsal sensillum *t1* filiform; *b* in row with *c*; *d* close to *c*; *e* nearer to *f* than to *d.* Abd. app. II–III each with two setae, the apical one nearly half the length of the subapical. Striate band on abd. VIII reduced, with no distinct striae (but a minute serration is present near its anterior edge). Seta *P3* on Tg. II–VI anterior to other *P*-setae row. St. VIII with 4/0 setae. All the tergal accessory setae sensilliform. Female squama genitalis with pointed acrostyli.

Type species. *Chosonentulus chosonicus* Imadaté & Szeptycki, 1976.

Distribution. Korea and China (after Wu and Yin 2008).

Remarks. For figures, see Imadaté and Szeptycki (1976), Wu and Yin (2008).

###### 22 – *Delamarentulus* Tuxen, 1963

Rostrum slightly developed. Labial palps reduced to an apical and two lateral setae and a setiform curved sensillum. Sensilla of maxillary palps setiform. Canal of maxillary glands with long and slender calyx; the proximal part long. Pseudoculi with divided lid; posterior prolongation very small. All foretarsal sensilla present; *t3* very long, lancet-like; *t1* claviform [in *D. pachychaetus* Tuxen and *D. tristani* (Silvestri) the distal half bent forwards in a rather sharp angle]. Abd. app. II–III each with an apical seta nearly 1/3^rd^ the subapical length. St. I–VII with three *A*-setae; St. VIII with 4/0 setae. Striate band complete. Comb VIII slightly concave with teeth of medium length. Female squama genitalis prolonged with very long, slender and pointed acrostyli.

Type species. *Acerentulus tristani* Silvestri, 1938.

Distribution. Tropical America and Africa.

Remarks. Figures and a key to the species are available in Tuxen (1979).

###### 23 – *Gracilentulus* Tuxen, 1963

Labial palps reduced to three setae and a small elongated sensillum. Maxillary palps with two sensilla. Canal of maxillary glands with rather large heart-shaped calyx. Pseudoculi small, broader than long. Foretarsi with small knob-like sensillum *t3* (except in *G. sarmaticus* Shrubovych and Szeptycki where *t3* is large, subequal in length to *t1*); *t1* claviform; *a* and *a*’ usually broader than the other sensilla (in *G. sarmaticus* *b* is broad and long as in *Podolinella*); *b*’ present or absent. Abd. app. II–III each with two setae, a median apical one very short, often only 1/5^th^ of the subapical. Seta *P3* on Tg. II–VI placed anteriorly to the row of the other *P*-setae. St. I–VII with three anterior setae. St. VIII with 4/0 or 4/2 (rarely) setae. Striate band well developed. Female squama genitalis with long pointed acrostyli.

Type species. *Acerentulus gracilis* Berlese, 1908.

Distribution. Cosmopolitan.

Remarks. Figures and a key to the species of *gracilis* group are available in Szeptycki (1993).

###### 24 – *Kenyentulus* Tuxen, 1981

Labial palps reduced to three setae and a sensillum. Calyx smooth and proximal canal of maxillary glands with two or three beady widenings. Sensillum *t1* on foretarsi baculiform; *b* very short. Metanotum with a small *P2a* seta quite close to *P3*. Abd. app. II–III each with a short median apical seta and a long subapical one. Seta *P1a* on Tg. II–VI very close to *P1*. St. VIII with 4/0 setae. Striate band reduced, with striae only faintly visible using phase contrast. Comb VIII with small pointed teeth. Acrostyli of female squama genitalis pointed.

Type species. *Acerentulus kenyanus* Condé, 1948.

Distribution. East and Tropical Asia; one species (*K. kenyanus*) pantropical.

Remarks. For figures, see Imadaté (1991), Yin (1999).

###### 25 – *Madagascaridia* Nosek, 1978

Labial palps reduced to two setae and a sensillum. Canal of maxillary glands with heart-shaped calyx and three lobes on the distal part near calyx, proximally deeply bipartite. All foretarsal sensilla present; *c* and *d* close to each–other. Abd. app. II–III each with one seta only. Striate band on abd. VIII reduced, with strongly dispersed striae. Comb VIII with small pointed teeth. Female squama genitalis with long pointed acrostyli.

Type species. *Madagascaridia condei* Nosek, 1978.

Distribution. China, Madagascar.

Remarks. For figures, see Nosek (1978b) and Yin (1999).

###### 26 – *Maderentulus* Tuxen, 1963

Maxillary and labial palps with tuft of setae and sausage-like sensilla (two and one, respectively). Canal of maxillary glands with long and slender calyx and short proximal part; the terminal dilation as in *Acerentulus*. Pseudoculi small. Foretarsal sensillum *t3* bacilliform; *t1* claviform; *b*, *c* and *d* in a row at the same level. Abd. app. II–III each with two setae, the median apical one more than half the length of the subapical. Tg. II–VI with seta *P3* in anterior position to *P*-setae row. St. VIII with 4/2 setae. Striate band well developed. Comb VIII with toothed margin. Female squama genitalis tripartite.

Type species. *Acerentulus maderensis* Condé, 1957.

Distribution. Iberian Peninsula, Macaronesia, Serbia (Blesic and Mitrovski–Bogdanović, 2012).

Remarks. For figures, see Tuxen (1982).

###### 27 – *Najtentulus* Szeptycki & Weiner, 1997

Sensilla of maxillary palps thick; labial palps with tuft of setae and a thick sensillum. Maxillary glands with large, smooth, nearly round calyx and short posterior filament. Foretarsal sensillum *b*’ present; *t1* fusiform; *t3* long, cylindrical; *d* situated proximally to the level of *t2*, nearer to *c* than to *e*; all sensilla of the inner side of foretarsi thick; setae *β1* and *δ4* short, sensilliform. Meso- and metanotum with seta *P2a* nearer to *P3* than to *P2*; setae *P1a*, *P2a* and *P5* oblong microchaetae; *P4a* on metanotum short microchaeta. Abd. app. II–III each with three setae, the lateral apical approx. twice as long as the median one. Seta *P3* on Tg. II–VI anterior to line *P2*–*P4*. Laterotergite VIII with peculiar, circular structure in anterior part. Tg. X with seta *P1a* present. St. VIII with 4/2 setae. Striate band on abd. VIII well developed, broad. Hind margin of segments IX–XII smooth. Female squama genitalis with apically situated acrostyli.

Type species. *Najtentulus silvestris* Szeptycki & Weiner, 1997.

Distribution. West Europe.

Remarks. For figures, see Szeptycki and Weiner (1997).

###### 28 – *Neobaculentulus* Yin, 1984

Maxillary palps with two sensilla: one setiform, the other torch light-shaped. Labial palps reduced to two setae and a sensillum. Calyx of maxillary glands simple. Foretarsi with all the sensilla; *a* to *g* and *a*’ to *c*’ present; *t1* baculiform; *t3* short (lanceolate). Abd. app. II–III each with two setae, a long subapical and a short median apical one. Tg. II–VI often with seven pairs of *P*-setae, seta *P1a* often absent; nine pairs of *P*-setae on Tg. VII. St. VIII with 4/0 setae. Striate band reduced, the striae invisible. Comb VIII oblique with small teeth. Female squama genitalis with pointed acrostyli.

Type species. *Berberentulus izumi* Imadaté, 1965.

Distribution. Far East.

Remarks. For figures and a key to species, see Bu and Xie (2006).

###### 29 – *Notentulus* Yin, 1989

Labial palps without tuft, with two to four setae and one sensillum. Maxillary glands with heart-shaped calyx with lobed distal excrescences; proximal part without dilations. Foretarsal sensillum *t1* baculiform; *b*’ absent. Abd. app. II–III each with two setae, the lateral apical one half the length of the subapical. Tg. VII with *Ac* seta. St. VIII with 4/0 setae. Striate band with short, irregular striae, and arranged in a waved line. Comb oblique and slightly concave, with sparsely sharp teeth. Female squama genitalis with pointed acrostyli.

Type species. *Notentulus zunyinicus* Yin, 1989.

Distribution. China, Central America.

Remarks. For figures, see Yin (1999).

###### 30 – *Podolinella* Szeptycki, 1995

Labial palps reduced to two lateral setae, a single terminal tuft-seta and a small claviform or oval sensillum. Maxillary glands with smooth calyx and bilobate posterior dilation. Foretarsi with a relatively large leaf-like sensillum *t3*; *b* large, passing the empodium. Notal setae short. Prosternum without seta *A2*. Abd. app. II–III each with two setae, the median apical seta less than half the length the subapical one. Seta *P3* on Tg. II–VI anterior to line *P2*–*P4*. St. I‒VII with three *A*-setae. St. VIII with 4/0 setae. Striate band complete. Comb VIII with 7–12 small teeth.

Type species. *Podolinella podolica* Szeptycki, 1995.

Distribution. Europe (after Galli et al. 2016).

Remarks. For figures, see Szeptycki (1995b).

###### 31 – *Polyadenum* Yin, 1980

Rostrum short. Labial palps reduced to three setae and a thick sensillum. Proximal part of the canal of maxillary glands three-branched. Foretarsal sensillum *t1* claviform; *b*, *c* and *d* nearly aligned; *f* halfway between *e* and *g*; *a*’ thick, situated between *t1* and *t2*; *b*’ missing. Abd. app. II–III each with two setae, the apical one approx. half the length of the subapical. St. VIII with 4/0 setae. Striate band on abd. VIII reduced, with indistinct striae. Female squama genitalis with tripartite perigynium and pointed acrostyli.

Type species. *Polyadenum sinensis* Yin, 1980.

Distribution. China.

Remarks. For figures, see Yin (1999).

###### 32 – *Proacerella* Bernard, 1975

Labial palps reduced to four setae and a sensillum. Distal end of canal of maxillary glands bulbous; faint expansions proximal to the calyx. Foretarsal sensillum *t1* claviform; *g* nearly at the same level of *t3* and of approx. the same length; *b*’ absent. Abd. app. II–III each with two setae, the lateral apical one more than half the subapical. St. VIII with 4/0 setae. Striate band well developed, with hook-shaped design in *P. reducta* Bernard (after Tuxen, 1981). Comb VIII with approx. 12 teeth. Female squama genitalis with pointed stylus, passing the apex of acrostyli.

Type species. *Proacerella reducta* Bernard, 1975.

Distribution. North America, West Europe.

Remarks. For figures, see Bernard (1975), Aldaba (1983).

###### 33 – *Silvestridia* Bonet, 1942

Labial palps reduced to two or three setae and a sensillum. Sensillum of maxillary palps willow leaf-shaped. Canal of maxillary glands with heart-shaped calyx and short or longer proximal part not exceeding the proximal branch of the fulcrum; more or less bipartite in the proximal part. Pseudoculi circular with fork-shaped prolongation. All sensilla present on foretarsi; *t1* claviform; *t3* willow leaf-shaped; *a*’ shaped as an old Roman vase, close to *t1*; *b* spindle-shaped, reaching seta *γ4*. Seta *P3* on Tg. II–VI is slightly anterior to the other *P*-setae. Abd. app. II–III each usually with one seta only, the subapical one (rarely also a delicate median apical seta is present). St. I–VII with three *A*-setae; St. VIII with 4/0 setae. Striate band on abd. VIII reduced, with distinct dispersed striae proceeding from the proximal border. Comb VIII concave with small teeth. Female squama genitalis with relatively short apodeme and long pointed acrostyli.

Type species. *Silvestridia artiochaeta* Bonet, 1942.

Distribution. Pantropical.

Remarks. For figures, see Tuxen (1980), Yin and Dallai (1985).

###### 34 – *Tasmanentulus* Tuxen, 1985

Labial palps reduced to four setae and a sensillum. Canal of maxillary glands with heart-shaped calyx, sometimes with distal dilations of variable size and shape. Sensillum *t1* on foretarsi claviform; *t3* more or less parallel-sided; *d* closer to *c* than to *e*; *b*’ present or absent. Abd. app. II–III each with two setae, the lateral apical one more than half as long as the subapical. Striate band partly reduced (the striae may be seen more or less distinctly, but never as distinct) with hook-shaped design. St. VIII with 4/2 setae. Comb VIII with fairly large teeth. Female squama genitalis with small pointed acrostyli.

Type species. *Gracilentulus tasmanicus* Tuxen, 1967.

Distribution. Australia, New Zealand.

Remarks. For figures and a key to species, see Tuxen (1986).

###### 35 – *Tuxenidia* Nosek & Cvijović, 1969

Labial palps reduced to three setae and remnant of terminal tuft, composed of a bifurcate seta. Maxillary palps of medium length; sensilla equal, long, thin, almost setiform. Canal of maxillary glands with oval, smooth calyx. Foretarsi with baculiform sensillum *t1*; *t*3 long, nearly cylindrical; *e* closer to *f* than to *d; c* halfway between *d* and *b*; *b*’ present; setae *β1* and *δ5* shorter than other setae of “*δ*” group, resembling sensilla; *δ5* proximal to *c*’. Setae *P1a* and *P2a* on meso- and metanotum developed as gemmate microchaetae. Prosternum with two *A*-setae (*A*2 present). Abd. app. II–III each with one or two setae. Seta *P3* on Tg. II–VI anterior to other *P*-setae line. Accessory setae on abd. II–VII shorter than 1/5^th^ the length of the main setae, setiform on both Tg. and St. Striate band on abd. VIII well developed, with strong striae and distinct, granulated anterior line. Comb VIII with convex hind margin. Hind margin of segments IX–XII smooth. Female squama genitalis with knob-like acrostyli.

Type species. *Tuxenidia balcanica* Nosek & Cvijović, 1969.

Distribution. Balkans, Near East.

Remarks. For figures and comparative diagnosis of *Tuxenidia* spp., see Szeptycki and Broza (2004).

###### 36 – *Vindobonella* Szeptycki & Christian, 2001

Labial palps without tuft, with three setae and one long, thick, parallel-sided sensillum. Calyx of maxillary glands ovoid, smooth. Head with *sd4* seta. Foretarsi with sensillum *b*’; *d* nearer to *c* than to *e*; *t1* claviform; *t2* spindle-like; *t3* cylindrical. Seta *P2a* on meso- and metanotum nearer to *P3* than to *P2*. Abd. app. II–III each with a median apical seta less than half the length of the subapical. Seta *P3* on Tg. II–VI anterior to line *P2*–*P4*. St. VIII with 4/0 setae. Striate band on abd. VIII well developed. Comb VIII with straight hind margin and nearly ten slender teeth. Female squama genitalis short, with short subapical bidentate acrostyli.

Type species. *Vindobonella leopoldina* Szeptycki & Christian, 2001.

Distribution. Austria.

Remarks. For figures, see Szeptycki and Christian (2001).

###### 37 – *Yinentulus* Tuxen, 1985

Labial palps reduced to three setae and a sausage-shaped sensillum. Maxillary palps with tuft of setae and two pointed sensilla. Canal of maxillary glands with heart-shaped calyx, distal extensions and the proximal part less than half as long as proximal branch of fulcrum. The head in profile has a prominent “front”. All foretarsal sensilla present; *t1* baculiform; *t3* expanded distally; sensillum *b* distal to *c–d*, extremely long. Abd. app. II–III each with two setae, the lateral apical one shorter than the subapical. Striate band on abd. VIII reduced. Comb VIII with many very small teeth. Female squama genitalis with pointed acrostyli.

Type species. *Yinentulus paedocephalus* Tuxen, 1986.

Distribution. New Zealand.

Remarks. For figures, see Tuxen (1986a).

###### 38 – *Zangentulus* Yin, 1983

Labial palps reduced to two setae and a sensillum. Canal of maxillary glands with smooth and simple kidney bean-shaped calyx; end of proximal canal slightly tripartite. Pseudoculi circular. Foretarsal sensillum *t1* claviform; *b*’ present; all the exterior sensilla extremely long. Abd. app. II–III each with two setae, the median apical one less than half the length of the subapical. Striate band on abd. VIII well developed, with striae slightly varied in shape and length. Comb VIII with straight posterior margin. Female squama genitalis with pointed acrostyli.

Type species. *Zangentulus sinensis* Yin, 1983.

Distribution. China.

Remarks. For figures, see Yin (1999).

##### Subfamily Acerentominae Silvestri, 1907

Maxillary palps with apical tuft of setae and two basal sensilla. Labial palps generally well developed with tuft of setae, two or three distinct setae and one sensillum. Meso- and metanotum each with two pairs of *A*-setae, or three pairs of *A*-setae on the mesonotum and four on the metanotum. Striate band well developed or reduced.

###### 39 – *Acerentomon* Silvestri, 1907

Labrum almost always prolonged into a rostrum. Labial palps with tuft of setae, three setae and one sensillum lancet-like or knife-shaped. Calyx of maxillary glands simple, small; short proximal part. Foretarsal sensillum *t1* claviform; *t2* long, setiform; *t3* leaf-like, large; *b*’ absent; *a*’ displaced distally, close to *c*’ and *δ5*; *f* and *g* close to each other; *b* and *c* at the same level; very long seta *δ4* which contrasts with the length of other *δ*-setae. Three pairs of *A*-setae (*A2*, *A3*, *A4*) on the mesonotum and four pairs (*A2*, *A3*, *A4*, *A5*) on the metanotum. Abd. app. II–III each with two setae (the median apical seta less than half the length of the subapical one) or with only the subapical seta in one case (*A. aceris* Rusek). Seta *P3* on Tg. II–VI on the same row of the other *P*-setae. St. VIII with 4/0 or 4/2 setae. Striate band on abd. VIII complete with distinct striae. Comb VIII with in most cases 12–14 long and strong teeth. Pleural pectines present on segments VI–VII and, exceptionally, on V. Seta *x* sometimes present on Tg. VII between *A4* and *P4*. Female squama genitalis with long, pointed acrostyli (bipartite in *A. balcanicum* Ionescu).

Type species. *Acerentomon doderoi* Silvestri, 1907.

Distribution. West Palearctic.

Remarks. Figures and a recent key to the species belonging to the “*doderoi*” group are given by Shrubovych et al. (2016).

###### 40 – *Filientomon* Rusek, 1974

Labial palps with tuft of setae, two setae and one foliaceous sensillum. Canal of maxillary glands with a racemose appendix on the calyx. Pseudoculi broader than long. Foretarsal sensillum *t1* filiform; *t2* setiform; *t3* lanceolate; *a*’ between *t1* and *t2*; not modified setae *β1* and *δ4.* Three pairs of *A*-setae (*A2*, *A3*, *A4*) on mesonotum and four pairs of *A*-setae (*A2*, *A3*, *A4*, *A5*) on metanotum. Abd. app. II–III each with two setae of equal length (lateral apical and subapical) or with only the subapical seta in *F. qianshanense* Bu & Xie. Seta *P3* on Tg. II–VI in line with other *P*-setae. St. VIII with 4/0 setae. Striate band well developed. Comb VIII with 10-20 teeth of slightly irregular size; the posterior margin rounded, distinctly protruding backward. Abd. II–VIII with minute pleural pectines and weak rotary wheels. Micro-teeth (often indistinct) along the hind margin of Tg. VIII–XI. Female squama genitalis with long pointed acrostyli.

Type species. *Acerentulus lubricus takanawanus* Imadaté, 1956.

Distribution. Far East, Siberia, North America.

Remarks. For figures and a key to species, see Bu and Xie (2007).

###### 41 – *Fjellbergella* Nosek, 1978

Canal of maxillary glands similar to that of *Acerentomon*. Foretarsal sensillum *t1* spatulate; *t3* finger-shaped; *b* and *c* on different levels; *d* near to *t2*; *b*’ present. Meso- and metanotum each with two pairs of *A*-setae (*A2*, *A4*). Abd. app. II–III each with three setae different in length. Seta *P3* on Tg. II–VI situated in anterior position. St. VIII with 4/2 setae. Striate band on abd. VIII reduced as in *Berberentulus*. Comb VIII rectangular with 10 slender teeth. Female squama genitalis with long pointed acrostyli.

Type species. *Fjellbergella tuxeni* Nosek, 1980.

Distribution. Alaska, Russian Far East (after Bu et al. 2014).

Remarks. For figures, review and a key to species, see Shrubovych and Bernard (2013) and Bu et al. (2014).

###### 42 – *Huashanentulus* Yin, 1980

Labial palps with tuft of setae, three distinct setae and a large sensillum. Maxillary glands of the *Acerentulus*-type, with a smooth, simple enlarged calyx; the proximal part long, with a terminal dilation. Pseudoculi circular, enclosed in an elliptical cavity. Foretarsal sensillum *t1* filiform; *t1*, *b* and *c* at the same level; *d* distal to *t2*; *e* near to *d*; *a*’ between the level of *t1* and *t2*; *b*’ missing; *c*’ proximal to *t3*. Meso- and metanotum each with two pairs of *A*-setae (*A2*, *A4*). Seta *P3* on Tg. II–VI situated anterior to other *P*-setae row. Abd. app. II–III each with two setae, the lateral apical one longer than half the subapical. St. I–VII with three *A*-setae. Striate band on abd. VIII well developed. Comb VIII slightly protruding backwards.

Type species. *Huashanentulus huashanensis* Yin, 1980.

Distribution. China.

Remarks. For figures and a key to species, see Bu and Yin (2010).

###### 43 – *Orinentomon* Yin & Xie, 1993

Labial palps with tuft of setae, two or three distinct setae and a thick sensillum. Canal of maxillary glands relatively short, the ellipsoidal calyx with smooth, elliptic appendix on its dorsal side. Foretarsal sensillum *t1* claviform; *t2* setaceous; *t3* lanceolate; *a*’ approx. at the same level of *t1*; *b*’ absent. Mesonotum with three pairs of *A*-setae (*A2*, *A3*, *A4*), metanotum with four pairs of *A*-setae (*A2*, *A3*, *A4*, *A5*). Abd. app. II–III each with two setae, the lateral apical one longer than half the subapical. Seta *P3* on Tg. II–VI in line with other *P*-setae. St. VIII with 4/2 setae. Striate band on abd. VIII well developed. Comb VIII with irregular teeth. Female squama genitalis with sharp acrostyli.

Type species. *Orinentomon sinensis* Yin & Xie, 1993.

Distribution. Alaska, China.

Remarks. For figures, see Nosek (1980) and Yin (1999).

###### 44 – *Sugaentulus* Imadaté, 1978

Labial palps with tuft of setae. Calyx of maxillary glands simple or slightly granulated with an extra appendix; posterior filament short with apical dilation. Foretarsal sensillum *t1* claviform; *t2* thin; *t3* small, lanceolate; *b* and *c* nearly at the same level; *d* fairly close to *e*, a bit proximal respect to seta *γ3*; *f* and *g* very close to each other; *a*’ fairly distal to *t1*, at approx. the same level of *t2*; *b*’ absent; seta *β1* short. Meso- and metanotum each with two pairs of *A*-setae (*A2*, *A4*). Abd. app. II–III each with two setae, the median apical one 2/3^rd^ the length of the subapical. Seta *P3* on Tg. II–VI a little anterior to the line of *P*-setae; all accessory setae very short, sensilliform. St. I–VII with three *A*-setae. St. VIII with 4/2 setae. Striate band on abd. VIII well developed, with distinct striae. Comb VIII with straight hind margin, not protruding posteriorly and nearly 20 small regular teeth. Female squama genitalis with stout apically bipartite acrostyli.

Type species. *Sugaentulus masumii* Imadaté, 1978.

Distribution. Japan, Russia (Siberia) (after Shrubovych and Rusek, 2010).

Remarks. For figures and the distinction between the two known species, see Shrubovych and Rusek (2010).

###### 45 – *Tuxenentulus* Imadaté, 1973

Labial palps with tuft of setae. Canal of maxillary glands with globular calyx; the proximal part relatively short. Foretarsal sensilla similar to those of *Acerentulus* both for shape and position; *a*’ close to *t2*; *b*’ present; *t1* claviform. Meso- and metanotum each with two pairs of *A*-setae (*A2*, *A4*). Abd. app. II–III each with two setae, the median apical one 2/3^rd^ the length of the subapical. Seta *P3* on Tg. II–VI slightly anterior to the other *P*-setae. Striate band on abd. VIII reduced as in *Berberentulus*. Comb VIII square, not oblique. Pleural pectines and rotary wheels on abd. present, but sometimes indistinct. Female squama genitalis with pointed acrostyli.

Type species. *Tuxenentulus ohbai* Imadaté, 1973.

Distribution. Northeast Asia, North America.

Remarks. For figures and a key to species, see Shrubovych and Bernard (2013).

###### 46 – *Wenyingia* Imadaté, 1986

Labial palps with tuft of setae, and a slender sensillum. Canal of maxillary glands simple, smooth, calyx not globular. Foretarsi with all sensilla; *t1* filiform; *t3* slender and rounded apically; *c* and *b* at approx. the same level; *d* closer to *c* than to *e*, a bit more proximal than *t2*; *a*’ slightly distal to *t1*; *b*’ present, at approx. the same level of *δ3*. Meso- and metanotum each with two pairs of *A*-setae (*A2*, *A4*). Abd. app. II–III each with two setae, the lateral apical one strong and longer than half the subapical. On Tg. II–VI seta *P3* a little anterior to the line of other *P*-setae; all accessory setae very short, sensilliform. St. I–VII with three *A*-setae; St. VIII with 4/2 setae. Striate band on abd. VIII reduced. Comb VIII not protruding posteriorly. Female squama genitalis stout, with pointed acrostyli.

Type species. *Yinentulus kurosawai* Imadaté, 1986.

Distribution. Japan.

Remarks. For figures, see Imadaté (1986b)

###### 47 – *Yamatentomon* Imadaté, 1964

Labrum slightly prolonged. Labial palps with tuft of setae and slender or broad sensillum. Canal of maxillary glands unbranched with heart-shaped smooth calyx and with a helmet-like dorsal appendix. Pseudoculi elevated on the surface, longitudinally divided with distinct posterior fork-shaped prolongation. Foretarsal sensillum *b* long; *d* placed halfway between *c* and *e* or closer to *e*; *f* and *g* very close to each other; *t1* baculiform; *t3* lancet-like; *a*’ long, close to *t1*; *b*’ absent. Three pairs of *A*-setae on the mesonotum (*A2*, *A3*, *A4*) and four pairs on the metanotum (*A2*, *A3*, *A4*, *A5*). Abd. app. II–III each with two setae (lateral apical seta slightly shorter than subapical one). Seta *P3* on Tg. II–VI in line with other *P*-setae. Tg. II–VII with eight or more *A*-setae; Tg. VIII with six *A*-setae. St. II–VII with five or more *A*-setae; St. VIII with 4/0 setae. Striate band on abd. VIII well developed. Comb VIII convex, with very fine teeth. Posterior border of abd. VIII–IX with very fine teeth. Pleurite VIII with lateral pectines. Female squama genitalis with long pointed acrostyli.

Type species. *Acerentulus yamato* Imadaté & Yosii, 1956.

Distribution. Far East (Japan, Korea, northeastern China and Russian Far East).

Remarks. Figures and a key to species are available in Bu and Wu (2012).

###### 48 – *Yichunentulus* Yin, 1980

Labial palps with one-branched terminal tuft of setae, three setae and a sensillum. Maxillary glands simple and without appendages. Foretarsal sensillum *t1* baculiform; *t3* short, lanceolate; sensillum *b*’ absent or present; *a*’ located distal to *t1*. Meso- and metanotum each with two pairs of *A*-setae (*A2*, *A4*). Abd. app. II–III each with two setae, the median apical one approx. half the length of the subapical. Seta *P3* on Tg. II–VI anterior to other *P*-setae row. St. II–VI with three *A*-setae; St. VIII with 4/2 setae. Striate band on abd. VIII reduced as in *Berberentulus*. Comb VIII with 10-12 small teeth. Female squama genitalis with pointed acrostyli.

Type species. *Yichunentulus yichunensis* Yin, 1980.

Distribution. China.

Remarks. For figures and a key to species, see Bu et al. (2014).

###### 49 – *Liaoxientulus* Wu & Yin, 2011

Head without differentiated sensory setae. Postpseudocular (*sd4*) seta present. Labial palps reduced, with three setae and no sensillum. Canal of maxillary glands simple, with smooth, ovate calyx and long, simple posterior filament. Pseudoculi nearly circular. All foretarsal sensilla present; dorsal sensillum *t1* claviform; *t2* thin and pointed; *t3* broad jar-shaped; *c* and *d* close to each other; *f* distinctly nearer to *e* than to *g*; *a*’ slightly proximal to *t1*; *b*’ at approx. the same level of *δ3*; ventral seta *β1* and interior seta *δ4* both short and sensilliform. Meso- and metanotum each with two pairs of *A*-setae (*A2*, *A4*); seta *P2a* nearer to *P3* than to *P2*. Abd. app. II–III each with two setae, the lateral apical one more than half as long as the subapical. On Tg. II–VI seta *P3* slightly anterior to other *P*-setae row; accessory setae very short, sensilliform. St. I–VII with three *A*-setae; St. VIII with with 4/2 setae. Striate band on abd. VIII, not fully developed. Comb VIII with eight irregular teeth. Female squama genitalis with long pointed acrostyli.

Type species. *Liaoxientulus xingchengensis* Wu & Yin, 2011.

Distribution. China.

Remarks. For figures, see Wu and Yin (2011).

##### Subfamily Nipponentominae Yin, 1983

Labial palps fully developed with tuft of setae. Maxillary glands with two racemose appendices or a distinct vesicle on the calyx. Meso- and metanotum each with two pairs of *A*-setae, or three pairs of *A*-setae on the mesonotum and three or four pairs of *A*-setae on the metanotum. Abd. app. II–III each with two setae. Striate band on abd. VIII well developed.

###### 50 – *Alaskaentomon* Nosek, 1977

Labial palps with a sensillum. Canal of maxillary glands with verrucose appendices on calyx and helmet-like dilation on the cover. Foretarsal sensillum *a* extraordinarily long, reaching seta *γ4*; *t1* filiform. Meso- and metanotum each with two pairs of *A*-setae (*A2*, *A4*). Lateral apical and subapical seta on abd. app. II–III nearly of equal length. Tg. II–VI with eight *A*-setae. Seta *Pc* present on Tg. VII and St. VI–VII. St. VIII with 4/2 setae. Striate band on abd. VIII with fine serrated line anteriorly and well developed striae posteriorly. Comb VIII with 10-15 teeth.

Type species. *Alaskaentomon fjellbergi* Nosek, 1977.

Distribution. Alaska.

Remarks. For a recent revision of genus *Alaskaentomon*, see Shrubovych et al. (2014c).

###### 51 – *Callientomon* Yin, 1980

Labial palps with a broad sensillum. Canal of maxillary glands with enlarged calyx, decorated with radiate–rods appendices and an extra–appendix on the cover. Pseudoculi circular. Foretarsal sensillum *t1* filiform; *b*’ absent. Pronotum with three pairs of setae (*1*, *1*’, *2*). Meso- and metanotum each with three pairs of *A*-setae (*A2*, *A3*, *A4*). Lateral apical seta on abd. app. II–III longer than half the subapical. St. VIII with 4/2 setae. Comb VIII with curved hind margin and approx. 20 small, slender teeth. Female squama genitalis with pointed acrostyli.

Type species. *Callientomon chinensis* Yin, 1980.

Distribution. China.

Remarks. For figures, see Yin (1999) and Shrubovych (2014).

###### 52 – *Imadateiella* Rusek, 1974

Labial palps with two lateral setae and a foliaceous sensillum. Pseudoculi almost circular or broader than long. Canal of maxillary glands with racemose appendices on the calyx and, usually, an additional appendix on the cover. Foretarsal sensillum *t1* spindle-shaped; *t2* thin and long; *t3* lanceolate; *b*’ absent. Mesonotum with three pairs of *A*-setae (*A2*, *A3*, *A4*), metanotum with four pairs of *A*-setae (*A1*, *A2*, *A3*, *A4*). Mesosternum with five *A*-setae; metasternum with seven *A*-setae. Lateral apical seta on abd. app. II–III longer than half the subapical. Five pairs of *A*-setae on Tg. II–VI; four pairs of *A*-setae on Tg. VII. Seta *P3* on Tg. II–VI on the same row of the other *P*-setae. St. I–VII with three *A*-setae. St. VIII with 4/0 setae. Posterior margin of comb VIII not protruding backward. Abdominal pectinated structures as pleural pectines visible, but often indistinct. No teeth on the hind margin of St. IX–XI. Female squama genitalis with long pointed (or bipartite) acrostyli.

Type species. *Acerella shiria* Imadaté, 1964.

Distribution. East and Central Asia.

Remarks. Figures and a recent key to species are available in Shrubovych et al. (2015).

###### 53 – *Nienna* Szeptycki, 1988

Maxillary palps with basal sensilla equal, thin, nearly setiform. Labial palps with a broad sensillum. Calyx of maxillary glands with small granulated appendices. Pseudoculi circular, with short lever. Foretarsal sensillum *b*’ missing; *t1* filiform; *t3* globular; *b* and *c* nearly at the same level; *d* nearer to *e* than to *c*; *a*’ more proximal than *t2*; setae *β1* and *δ4* of normal shape, subequal to setae *δ1–δ3*. Meso- and metanotum each with three pairs of *A*-setae (*A2*, *A3*, *A4*); seta *P2a* on nota nearer to *P3* than to *P2*. Prosternum without seta *A2*. Lateral apical and subapical seta on abd. app. II–III nearly of equal length. Seta *P3* on Tg. II–VI in line with other *P*-setae. St. VIII with 4/2 setae. Hind margin of comb VIII straight. St. IX–X with traces of striate band. Hind margin of Tg. X–XI with subtle serration. Female squama genitalis with short, pointed acrostyli.

Type species. *Nienna parvula* Szeptycki, 1988.

Distribution. Central Asia, Northwest China (after Bu & Yin, 2008).

Remarks. For figures, see Szeptycki (1988).

###### 54 – *Nipponentomon* Imadaté & Yosii, 1959

Labrum prolonged into a short rostrum. Labial palps with three setae and a foliaceous sensillum. Canal of maxillary glands with verrucose calyx, often produced into side-flaps, the proximal part usually long and with long proximal dilation. Pseudoculi small, usually, broader than long. Foretarsal sensillum *t1* long and spindle-shaped; *t3* long, lancet-like; *d* close to *e*; *b* and *c* subequal in length; *a*’ a little bit more distal than *t2*; *b*’ missing. Mesonotum with three pairs of *A*-setae (*A2*, *A3*, *A4*); metanotum with four pairs of *A*-setae (*A1*, *A2*, *A3*, *A4*). Lateral apical and subapical seta on abd. app. II–III of equal length. Tg. I–VI with 8–10 *A*-setae. Seta *P3* on Tg. II–VI on the same row of the other *P*-setae. St. I–VI with three to five *A*-setae; VII with three *A*-setae; VIII with 4/2 setae. Comb VIII with approx. ten long and strong teeth. Posterior margin of abd. IX–XI with pectinated structures. Pleural pectines often present on segments II–VIII. Female squama genitalis with mostly square acrostyli with uneven apex or produced into three teeth; sometimes, long and pointed acrostyli.

Type species. *Acerentomon nippon* Yosii, 1938.

Distribution. Northeast Asia, North America.

Remarks. For a key to species, see Bu et al. (2013).

###### 55 – *Noldo* Szeptycki, 1988

Maxillary palps with equal, thin and nearly setiform basal sensilla. Labial palps with broad leaf-like sensillum. Calyx of maxillary glands small, granulated; posterior filament with granulated thikening. Pseudoculi circular, with relatively long lever. Foretarsal sensillum *b*’ missing; *a*’ slightly more proximal than *t2*; *t1* filiform; *t3* leaf-like; *b* and *c* nearly at the same level; *d* nearer to *e* than to *c*; setae *β1* and *δ4* sensillum-shaped. Meso and metanotum each with three pairs of *A*-setae (*A2*, *A3*, *A4*); seta *P2a* on nota nearer to *P3* than to *P2*. Prosternum with seta *A2* present. Lateral apical and subapical seta on abd. app. II–III nearly of equal length. Seta *P3* on Tg. II–VI in line with other *P*-setae. St. VIII with 4/2 setae. Comb VIII with straight hind margin. Abd. IX–X without striate band. Hind margin of Tg. X–XI with very subtle serration. Female squama genitalis with relatively short, pointed acrostyli.

Type species. *Noldo submontanus* Szeptycki, 1988.

Distribution. Russia and Ukraine (Crimea).

Remarks. For figures and the distinction between the two known species of genus *Noldo*, see Shrubovych and Szeptycki (2006).

###### 56 – *Nosekiella* Rusek, 1974

Labial palps with a broad sensillum. Canal of maxillary glands with two racemose appendices on the calyx and an additional appendix on the cover. Pseudoculi broader than long. Foretarsi with baculiform sensillum *t1*; *t2* long, setiform; *t3* lanceolate; *a*’ at the same level of or anterior to *t2* base. Meso- and metanotum each with three pairs of *A*-setae (*A2*, *A3*, *A4*). Lateral apical and subapical seta on abd. app. II–III nearly of equal length. Seta *P1a* present on Tg. I–VI. Tg. II–VI with 10 *A*-setae; Tg. VIII with four or six *A*-setae. Prosternum with or without seta *M2*. St. III with five *P*-setae; St. I–VII with three *A*-setae; St. VIII with 4/0 or 4/2 setae. Pleural pectines visible. Hind margin of segments IX–XI smooth. Female squama genitalis short, with blunt, bilobed acrostyli.

Type species. *Acerentulus danicus* Condé, 1947.

Distribution. Palearctic (after Shrubovych et al., 2014).

Remarks. For a recent revision of genus *Nosekiella*, see Shrubovych et al. (2014a).

###### 57 – *Paracerella* Imadaté, 1980

Maxillary and labial palps with tuft of setae and two and one sensilla, respectively. Maxillary glands with distinct calyx with racemose appendices on its surface. Foretarsal sensillum *t1* filiform; sensillum *d* placed at approx. halfway between *c* and *e* or a little closer to *e*; *a*’ fairly distal to *t1*, at approx. the same level of *t2*. Meso- and metanotum each with three pairs of *A*-setae (*A2*, *A3*, *A4*). Subapical seta on abd. app. II–III slightly longer than lateral apical. Seta *P3* on Tg. II–VI in line with other *P*-setae. St. I–VII with three *A*-setae. St. VIII with 4/0 or 4/2 setae. Comb on abd. VIII with irregular teeth.

Type species. *Acerella shirataki* Imadaté, 1964.

Distribution. China (after Bu et al., 2016), Japan and North America.

Remarks. Figures and a key to species are provided in Shrubovych and Smykla (2012).

###### 58 – *Verrucoentomon* Rusek, 1974

Labial palps with a four-branched terminal tuft and a sensillum. Maxillary glands with racemose surface of calyx and without wide racemose appendices. Foretarsal sensillum *t1* filiform; *t3* leaf-like; *d* near *e*; *a*’ at the level or distal to the base of *t2*; seta *β1* setiform; *δ4* short and blunt. Meso- and metanotum each with three pairs of *A*-setae (*A2*, *A3*, *A4*). Lateral apical and subapical seta on abd. app. II–III nearly of equal length. Five pairs of *A*-setae on Tg. II–VI; seta *P3* in line with other *P*-setae. St. VIII with 4/0 or 4/2 setae. Comb on abd. VIII with distinct teeth.

Type species. *Acerella kawakatsui* Imadaté, 1964.

Distribution. Central and East Asia, Central Europe, North America.

Remarks. Figures and a key to species are provided in Shrubovych and Bernard (2012). For a recent review of the North American species, see Shrubovych et al. (2015).

###### 59 – *Nanshanentulus* Bu & Yin, 2007

Maxillary palps with two sensilla, parallel-sided and equal in length. Labial palps with tuft of setae and a broad sensillum. Calyx of maxillary glands with two lateral racemose appendices and with posterior filament slightly split into two leaves. Pseudoculi ovate. Foretarsi with no sensillum *b*’; *t1* claviform; *t3* small and lanceolate; *c* slightly lower than level of *b*; *d* nearer to *e* than to *c*; *c* and *e* short and broad; *a*’ at the level of *t1*; setae *β1* and *δ4* of normal shape. Claw with single inner flap. Meso- and metanotum with three pairs of *A*-setae (*A2*, *A3*, *A4*); seta *P2a* on nota near to *P3* than to *P2*. Prosternum with no seta *A2*. Subapical seta on abd. app. II–III slightly longer than lateral apical. Seta *P1a* on Tg. I–VI absent; seta *P3* on Tg. II–VI in posterior position. St. VIII with 4/0 setae. Striate band on Tg. IX and X absent. Comb on abd. VIII with straight hind margin. Female squama genitalis robust and with short blunt acrostyli.

Type species. *Nanshanentulus urumchiensis* Bu & Yin, 2007.

Distribution. China.

Remarks. For figures, see Bu and Yin (2007).

###### 60 – *Vesiculentomon* Rusek, 1974

Maxillary palps with two equally long spike-like sensilla; labial palps with broad sensillum. Extraordinarily large vesicle on the maxillary glands, weakly granulated along its proximal part. Pseudoculi circular. Foretarsal sensillum *t1* baculiform; *t2* setiform; *t3* leaf-like. Mesonotum with three pairs of *A*-setae (*A2*, *A3*, *A4*); metanotum with four pairs of *A*-setae (*A1*, *A2*, *A3*, *A4*). Lateral apical and subapical seta on abd. app. II–III nearly of equal length. Comb on abd. VIII with 14 very small teeth. Hind margins of Tg. IX–XI and St. IX–X laterally finely serrated. Female squama genitalis short, with apically bilobed acrostyli.

Type species. *Vesiculentomon marshalli* Rusek, 1974.

Distribution. North America.

Remarks. Genus was recently revised by Shrubovych et al. (2014a,b).

###### 61 – *Nosekientomon* Shrubovych, Rusek & Bernard, 2014

Maxillary palps with two sensilliform sensilla; labial palps with broad sensillum. Maxillary glands with two racemose appendices and a smooth globular vesicle on the calyx. Foretarsal sensillum *t1* claviform; position of *d* midway between sensilla *c* and *e*; position of foretarsal sensillum *a*’ slightly distal to level of *t2*. Meso- and metanotum each with three pairs of *A*-setae (*A2*, *A3*, *A4*). Subapical seta on abd. app. II–III slightly longer than lateral apical one. Seta *P3* on Tg. II–VI in line with other *P*-setae. St. I–VII with three *A*-setae; St. VIII with 4/2 setae. Comb on abd. VIII with 10–11 short teeth. Hind margins of abd. IX–XII smooth. Female squama genitalis short, with blunt bilobed acrostyli.

Type species. *Vesiculentomon ruseki* Nosek, 1977.

Distribution. North America.

Remarks. For figures, see Shrubovych et al. (2014b).

###### 62 – *Yavanna* Szeptycki, 1988

Maxillary palps with thin, nearly setiform basal sensilla. Labial palps with broad basal sensillum. Pseudoculi circular, with short lever. Calyx of maxillary glands simple, large, with small granulation. Foretarsi with claviform sensillum *t1*; *t3* leaf-like; *b* and *c* nearly at the same level; *d* nearer to *e* than to *c*; *a*’ more distal than *t2*; *b*’ absent; seta *β1* of normal shape, *δ4* sensillum-shaped. Meso- and metanotum each with three pairs of *A*-setae (*A2*, *A3*, *A4*); seta *P2a* on nota nearer to *P3* than to *P2*. Prosternum with or without seta *M2*. Subapical seta on abd. app. II–III slightly longer than lateral apical. Seta *P1a* present on Tg. I–VII or on Tg. VII only. Tg. II–VI with 10 anterior setae and seta *P3* in line with other *P*-setae; Tg. VIII with six *A*-setae (*A2, A4, A5*). St. I–VII with three *A*-setae; St. III with five or six *P*-setae; St. VII with or without seta *Pc*; St. VIII with 4/2 setae. Hind margin of comb on abd. VIII slightly convex. St. IX–X with distinct striate band. Hind margin of Tg. X–XI smooth. Female squama genitalis short, with blunt, bilobed, or three-lobed acrostyli.

Type species. *Yavanna altaica* Szeptycki, 1988.

Distribution. Central Asia, China.

Remarks. Figures and a key to species are given by Shrubovych et al. (2012).

##### Subfamily Acerellinae Yin, 1983

Labial palps with terminal tuft of setae, two setae and an oval or foliaceous sensillum. Canal of maxillary glands with lateral verrucose appendices on calyx. Meso- and metanotum each with three pairs of *A*-setae (*A2*, *A3*, *A4*). Abd. app. II–III each with two setae (lateral apical and subapical) subequal in length.

###### 63 – *Acerella* Berlese, 1909

Maxillary palps with two sensilla spindle-shaped or very slender willow leaf-shaped. Pseudoculi broader than long, longitudinally divided, with distinct fork-shaped posterior prolongation. Foretarsal sensillum *t1* long, filiform; *t3* lanceolate, but not pointed; *d* usually closer to *e* than to *c*; *f* extremely long; *b*, *g* and *a*’ broad; *b*’ missing. Seta *P3* on Tg. II–VI on the same row of the other *P*-setae. St. I–VII with three *A*-setae; St. VIII with 4/0 setae. Striate band on abd. VIII well developed. Comb VIII with distinct teeth. Female squama genitalis with rather long acrostyli.

Type species. *Acerentulus tiarneus* Berlese, 1908.

Distribution. West Palearctic.

Remarks. For figures, see Nosek (1973) and Shrubovych (2011).

### Order SINENTOMATA Yin, 1966

Abdomen with pectinated structures. Median setae absent on meso- and metanotum. Abd. app. I always two-segmented with terminal vesicle and four setae. Female squama genitalis with long acrostyli.

#### Family Fujientomidae Tuxen & Yin, 1982

Pale body. Spiracles absent. Foretarsi with additional sensillum *x* on the external side. Abd. app. all two-segmented with four setae and terminal vesicle. Comb VIII with small teeth.

##### 64 – *Fujientomon* Imadaté, 1964

Rostrum somewhat protruded. Maxillary palps with tuft of setae and two sensilla. Labial palps reduced with an apical thick seta and two basal setae. Canal of maxillary glands simple, ending with a long sac- or sausage-like dilation. Pseudoculi circular without lever. Foretarsal sensilla *b*’ and *c*’ absent; sensillum *t1* extremely broad; *t2* broad; *t3* long and slender; exterior sensilla *a* and *x* broad, foliaceous. Empodium long, a little shorter than claw. Seta *S* remarkably longer than claw. Metanotum with two or three pairs of *A*-setae (*A3* is very short, sensilliform, often absent). Tg. I–VII with seta *Ac.* St. VIII with 4/4 setae. Comb VIII with approx. 15 small teeth, and a few small teeth on its anterior margin. Female squama genitalis with rod-like acrostyli.

Type species. *Fujientomon primum* Imadaté, 1964.

Distribution. China, Japan.

Remarks. Redescription by Nakamura (2014) was followed.

#### Family Sinentomidae Yin, 1965

Body reddish-brown. Tracheal system present. Pseudoculi large with 7–13 transverse striae. Foretarsi with additional sensillum *c*” on the internal side. Abd. app. II–III uni-segmented, each with two setae, a long distal and a short proximal one. Basal part of the female squama genitalis bucket-like, with large and stout acrostyli.

##### 65 – *Sinentomon* Yin, 1965

Integument highly sclerotized, pigmented in reddish-brown and covered with many scale-like portions with serrated, posterior margins. Labial palps with two setae and apical tuft. Canal of maxillary glands indistinct. Pseudoculi with transverse striation. Foretarsi with nine long and slender dorsal setae (*α1–9*), three dorsal filiform sensilla (*t1*, *t2* and *t3*), nine ventral setae (*β1–9*), five external setae (*γ1–5*), seven external sensilla (*a–g*), six internal setae (*δ1–6*) and four internal sensilla (*a*’, *b*’, *c*’, *c*”). Meso- and metanotum each with three pairs of *A*-setae (*A1*, *A2*, *A4*). Abd. VIII with smooth combs covering openings of abdominal glands. Female squama genitalis with median palps and long and pointed acrostyli.

Type species. *Sinentomon erythranum* Yin, 1965.

Distribution. China, Japan, North Korea.

Remarks. For figures, see Yin (1999).

### Order EOSENTOMATA Yin, 1996

Tracheal system normally present on meso- and metanotum. Foretarsus with additional setae and sensilla (e.g. *x*, *y*, *z*, *f2*, *b’2*). Abdomen without pectinated structures. Three pairs of two-segmented abd. app., each with terminal vesicle and five setae. Female squama genitalis with processus sternalis and without acrostyles.

#### Family Eosentomidae Berlese, 1909

Eosentomata with spiracles on meso- and metanotum. Pseudoculi small.

##### Subfamily Isoentominae Yin, 1983

Foretarsal sensilla *e* (if present) and *g* short or long, spiniform or setiform (spatulate only in genus *Zhongguohentomon*).

###### 66 – *Isoentomon* Tuxen, 1975

Foretarsal sensilla *g* and *e* (when present) spiniform or short and setiform (never spatulate); sensillum *b1*’ present. Seta *P1a* on Tg. I–VI longer than seta *P1*. Female squama genitalis with “dissolved”, in most cases extremely short processus sternalis.

Type species. *Eosentomon atlanticum* Condé, 1947.

Distribution. Warmer areas of the whole world.

Remarks. For figures and a key to species, see Tuxen (1975).

###### 67 – *Osientomon* Nakamura, 2010

Foretarsal sensillum *e* absent; *g* short and setiform; *b1*’ absent. On meso- and metanotum two pairs of *A*-setae; *P2a* closer to *P2* than to *P3*. St. XII with 12 setae (a pair of small sensilliform *A*-setae). On female squama genitalis, processus sternalis S-shaped with caput duck’s head-like.

Type species. *Osientomon japonicum* Nakamura, 2010.

Distribution. Japan.

Remarks. For figures and a key to species, see Nakamura (2010).

###### 68 – *Madagascarentomon* Nosek, 1978

Maxillary palps with two distinct sensilla. Labial palps with tuft of setae and a sensillum. All foretarsal sensilla, except *t1*, setiform; sensilla *e* and *g* long. Empodium very long. Female squama genitalis with caput processus resembling a snake’s head; stylus more than twice as long as broad; filum long.

Type species. *Madagascarentomon condei* Nosek, 1976.

Distribution. Madagascar.

Remarks. For figures and a key to species, see Nosek (1978b).

###### 69 – *Zhongguohentomon* Yin, 1979

Integument highly sclerotized, earth-yellow coloured. Labial palps with tuft of setae, two distinct setae and a sausage-like sensillum. Central seta present on antero-dorsal surface of the head (similar to that of *Sinentomon*). Pseudoculi with a large triangular prolongation on the anterior margin. Foretarsal sensillum *t1* clavate; *t3* stout; *e* and *g* short, slightly spatulate; *b1*’ and *b2*’ present; *c*’ fairly long. Abd. VIII with a pair of rudimentary combs on abdominal glands. Pectinated patches on St. XI–XII. Female squama genitalis characterized by a simple stylus with a nipple-shaped processus on its posterior end and the incurvated processus filum.

Type species. *Zhongguohentomon magnum* Yin, 1979.

Distribution. China.

Remarks. For figures and a key to species, see Yin (1999).

##### Subfamily Eosentominae Berlese, 1909

Spiracles present. Foretarsal sensilla *e* and *g* both present and spatulate or clavate.

###### 70 – *Eosentomon* Berlese, 1908

Canal of maxillary glands without dilation. Pseudoculi lens-like. Foretarsi with accessory setae *α3*’, *β8*, *β9*, *δ3*’ and *δ4*’ and extra sensilla *f2*, *b2*’, *x*, *y*, *z* and, rarely, *c2*’; sensilla *e* and *g* spatulate. Empodium long. Meso- and metanotum each with two pairs of *A*-setae (*A2*, *A4*). Female squama genitalis often with duck’s head-shaped caput of processus sternalis.

Type species. *Eosentomon transitorium* Berlese, 1909.

Distribution. Cosmopolitan.

Remarks. For figures and keys to Asian species, see Imadate (1965), Yin (1999) and Nakamura (2010). For North American species, see Allen (2007). For a key to European species, see Shrubovych and Bernard (2018).

###### 71 – *Styletoentomon* Copeland, 1978

Labrum longer than mandibles and very narrow, forming a rostrum. Mandibles very long, extremely slender and sharp pointed (stylet-shaped). Canal of maxillary glands reaching prothorax. Foretarsal sensilla *e* and *g* clavate. Female squama genitalis with sharp-pointed processus sternalis and evenly bent giving a stoop-shouldered appearance.

Type species. *Eosentomon rostratum* Ewing, 1940.

Distribution. North America.

Remarks. For figures and diagnosis, see Copeland (1978).

##### Subfamily Anisentominae Yin, 1983

Spiracles present (often quite small). Foretarsal sensillum *e* absent; *g* always spatulate. Tg. II–VI with 10 or eight *A*-setae; eight or six *A*-setae on Tg. VII; St. VIII always with seven *P*-setae (2/7 or 0/7). Tg. X or XI of some species bearing a pair of peculiar setae or spines (*Anisentomon*).

###### 72 – *Anisentomon* Yin, 1977

Spiracles very small. Labial palps with tuft of setae. Foretarsal sensillum *g* stout and spatulate. Tg. II–VII with eight *A*-setae. Tg. VIII with 6/9 setae (*Pc* present). Tg. X or XI with a pair of large spines. St. VIII with 2/7 setae (*Pc* present). Comb with smooth margin on laterotergite VIII. Female squama genitalis of *Eosentomon* type.

Type species. *Eosentomon chinensis* Yin, 1965.

Distribution. China.

Remarks. Xiong et al. (2008) report a key to species of genus *Anisentomon*.

###### 73 – *Neanisentomon* Zhang & Yin, 1984

Foretarsal sensillum *t2* willow leaf-shaped; *f1* spindle-like; *b2*’ and *c*’ often absent; *g* stout and spatulate. Tg. VIII with 6/8 setae (*Pc* absent). St. VIII with 2/7 setae (*Pc* present). Female squama genitalis similar to that of *Eosentomon*.

Type species. *Neanisentomon guicum* Zhang & Yin, 1984.

Distribution. China.

Remarks. For figures and a key to species, see Bu & Yin (2011).

###### 74 – *Paranisentomon* Zhang & Yin, 1984

Foretarsal sensilla *f1* and *t2* setiform. Tg. VIII with 6/9 setae (*Pc* present). St. VIII with a single row of seven *P*-setae (0/7). Processus sternalis often S-shaped on female squama genitalis.

Type species. *Eosentomon tuxeni* Imadaté & Yosii, 1959.

Distribution. China and Japan.

Remarks. For figures and a key to species, see Yin (1999).

###### 75 – *Pseudanisentomon* Zhang & Yin, 1984

Foretarsal sensillum *g* long spatulate; *t2* willow leaf-shaped or setiform; *f1* spindle-shaped. Tg. VIII with 6/9 setae (*Pc* present). St. VIII with 0/7 or 2/7 setae. Processus sternalis often S-shaped on female squama genitalis.

Type species. *Anisentomon songkiangensis* Yin, 1977.

Distribution. China and Japan.

Remarks. For figures and a key to species, see Yin (1999).

#### Family Antelientomidae Yin, 1983

Eosentomata without open spiracles on meso- and metanotum. Sensilla on foretarsus similar to those of genus *Eosentomon*.

##### 76 – *Antelientomon* Yin, 1974

Tg. VIII with 6/9 setae. St. VIII with *Pc* seta (2/7 or 2/9). Female squama genitalis similar to that of *Eosentomon*.

Type species. *Antelientomon prodromi* Yin, 1974.

Distribution. China.

Remarks. For figures and a key to species, see Yin (1999).

### Key to genera

This key directly identifies genera, without intermediate steps to families and subfamilies (indicated in the diagnosis part) for greater convenience and to keep it valid regardless of the system followed. Parts concerning the Acerentominae and Nipponentominae genera are taken from Shrubovych and Rusek (2010) and Shrubovych et al. (2014b), respectively. The previous key for Acerentomidae genera by Tuxen (1984) was also used as a reference. For Protentomidae and Hesperentomidae genera, we referred to the key to subfamilies and genera by Tuxen and Yin (1982). Numbers in brackets after the names of genera indicate their position in the diagnostic section of this paper.

**Table:** 

1	Meso- and metanotum with tracheal system and spiracles	**2**
–	Spiracles absent	**13**
2	Pectinated structures on the abdomen present; pseudoculi transversally striated; abdominal appendages I two-segmented, with terminal vesicle and four setae, abdominal appendages II and III unisegmented with two setae	***Sinentomon* (65)**
–	Abdominal pectinated structures absent; not striated pseudoculi; abdominal appendages I–III two-segmented, each with five setae	**3**
3	Foretarsal sensillum *e* absent	**4**
–	Foretarsal sensillum *e* present	**9**
4	Tergites X or XI with large spines	***Anisentomon* (72)**
–	Tergites X–XI without spines	**5**
5	Foretarsal sensillum *g* long, spatulate	**6**
–	Foretarsal sensillum *g* not long and spatulate	**8**
6	Tergite VIII with 6/8 setae (*Pc* absent)	***Neanisentomon* (73)**
–	Tergite VIII with 6/9 setae (*Pc* present)	**7**
7	Foretarsal sensillum *f1* setiform	***Paranisentomon* (74)**
–	Foretarsal sensillum *f1* spindle-shaped	***Pseudanisentomon* (75)**
8	Foretarsal sensillum *b1*’ absent	***Osientomon* (67)**
–	Foretarsal sensillum *b1*’ present	***Isoentomon* (66 – part)**
9	Foretarsal sensillum *e* spatulate (or clavate)	**10**
–	Foretarsal sensillum *e* not spatulate	**12**
10	Labrum long and narrow (rostrum); stylet-shaped mandibles	***Styletoentomon* (71)**
–	Labrum and mandibles of normal shape	**11**
11	Pseudoculi with a large triangular prolongation on the anterior margin	***Zhongguohentomon* (69)**
–	Pseudoculi lens-like	***Eosentomon* (70)**
12	Seta *P1a* on tergites I–VI longer than *P1*	***Isoentomon* (66 – part)**
–	Seta *P1a* on tergites I–VI shorter than *P1*	***Madagascarentomon* (68)**
13	Three pairs of two-segmented abdominal appendages	**14**
–	Abdominal appendages III uni-segmented	**17**
14	Abdominal appendages with five setae; abdominal pectinated structures absent	***Antelientomon* (76)**
–	Abdominal appendages with four setae	**15**
15	Foretarsal sensilla *b*’ and *c*’ absent	**16**
–	Foretarsal sensilla *b*’ and *c*’ present	***Hesperentomon* (1)**
16	Canal of maxillary glands with heart-shaped calyx with verrucose appendices and a proximal dilation; pseudoculi with a large triangular prolongation in the proximal part	***Hinomotentomon* (4)**
–	Canal of maxillary glands simple, ending with a long sac- or sausage-like dilation; pseudoculi circular without prolongation	***Fujientomon* (64)**
17	Abdominal appendages II two-segmented	**18**
–	Abdominal appendages II uni-segmented	**23**
18	Foretarsi with an additional sensillum *c*”; pseudoculi “open” posteriorly	***Ionescuellum* (2)**
–	Foretarsi without additional sensillum *c*”; pseudoculi never “open” posteriorly	**19**
19	Foretarsal sensilla *b*’ and *c*’ missing, *t1* always present (setiform); abdominal appendages III with two or three setae	**20**
–	Foretarsal sensilla *t1* and *c* often missing, all the other sensilla always present; abdominal appendages III always with two setae	**22**
20	Pseudoculi with a short lever; sternites IV–V without *Pc* seta	***Neocondeellum* (6)**
–	Pseudoculi circular without lever; sternites IV–V with *Pc* seta	**21**
21	Abdominal appendages III with two setae	***Paracondeellum* (7)**
–	Abdominal appendages III with three setae	***Condeellum* (5)**
22	Metanotum with only one pair of *A*-setae (*A2*); *t1* usually and *c* always present; distinct teeth on comb on abdominal segment VIII	***Proturentomon* (9)**
–	Metanotum with two pairs of *A*-setae (*A2*, *A4*); *t1* always and *c* sometimes missing; teeth on comb on abdominal segment VIII indistinct or missing	***Protentomon* (8)**
23	Sternite VIII with 2/4 setae	***Huhentomon* (3)**
–	Sternite VIII never with 2/4 setae (normally with 4/0 or 4/2 setae)	**24**
24	Calyx of maxillary glands granulated or racemose and/or with racemose/granulated appendices, abdominal appendages II–III each with two setae	**25**
–	Calyx of maxillary glands not as above (when weakly verrucose, abdominal appendages II–III have three setae)	**40**
25	Metanotum with two pairs of *A*-setae	**26**
–	Metanotum with three or four pairs of *A*-setae	**27**
26	Labial palps reduced, with one broad sensillum and three setae	***Chosonentulus* (21)**
–	Labial palps not reduced with a terminal tuft of setae and one lateral sensillum	***Alaskaentomon* (50)**
27	Metanotum with three pairs of *A*-setae	**28**
–	Metanotum with four pairs of *A*-setae	**37**
28	Foretarsal sensillum *t1* filiform	**29**
–	Foretarsal sensillum *t1* baculiform or claviform	**34**
29	Pronotum with three pairs of setae; maxillary glands with large vesicle bearing finger-like papillae	***Callientomon* (51)**
–	Pronotum with two pairs of setae; maxillary glands without papillae	**30**
30	Calyx of maxillary glands with small granulated appendices; foretarsal sensillum *t3* globular	***Nienna* (53)**
–	Calyx of maxillary glands not as the one of *Nienna*; foretarsal sensillum *t3* foliate	**31**
31	Foretarsal seta *β1* sensilliform	***Noldo* (55)**
–	Foretarsal seta *β1* setiform	**32**
32	Bases of sensilla *d* and *a*’ level with base of sensillum *t2* on foretarsi	***Paracerella* (57)**
–	One or both of the bases of sensilla *d* and *a*’ not at the same level of sensillum *t2* on foretarsi	**33**
33	Bases of sensilla *d* and *a*’ proximal to the base of *t2* on foretarsi	***Acerella* (63)**
–	One or both of the bases of sensilla *d* and *a*’ distal to base of sensillum *t2* on foretarsi	***Verrucoentomon* (58)**
34	Foretarsal sensillum *t1* baculiform	***Nosekiella* (56)**
–	Foretarsal sensillum *t1* claviform	**35**
35	Maxillary glands with smooth globular vesicle near calyx; foretarsal sensillum *d* central between sensilla *c* and *e*; seta *1* on sternites VIII and X short, one-third length of seta *2*	***Nosekientomon* (61)**
–	Maxillary glands without vesicle near calyx; foretarsal sensillum *d* close to *e*; seta *1* on sternite VIII longer than half the length of seta *2*	**36**
36	Foretarsal sensillum *a*’ distal to *t2*	***Yavanna* (62)**
–	Foretarsal sensillum *a*’ nearly at the same level of *t1* (proximal to *t2*)	***Nanshanentulus* (59)**
37	Foretarsal sensillum *t1* baculiform; maxillary glands with large vesicle near calyx	***Vesiculentomon* (60)**
–	Foretarsal sensillum *t1* filiform (setiform or spindle-shaped); maxillary glands without vesicle near calyx	**38**
38	Sternite VIII with 4/2 setae	***Nipponentomon* (54)**
–	Sternite VIII with 4/0 setae	**39**
39	Lateral apical and subapical setae on abdominal appendages II–III of equal length (in one case only the subapical seta present); comb VIII with the posterior margin rounded and protruding backward	***Filientomon* (40)**
–	Lateral apical seta on abdominal appendages II–III more than half the length of the subapical; posterior margin of comb VIII not protruding backward	***Imadateiella* (52)**
40	Abdominal appendages II–III each with one seta (rarely also a delicate median apical seta is present)	**41**
–	Abdominal appendages II–III each with two or three setae	**45**
41	Labial palps not reduced	***Acerentomon* (39 – part)**
–	Labial palps reduced	**42**
42	Striate band well developed	***Tuxenidia* (35 – part)**
–	Striate band reduced	**43**
43	Canal of maxillary glands with heart-shaped calyx and three lobes on the distal part near calyx, proximally deeply bipartite	***Madagascaridia* (25)**
–	Canal of maxillary glands without lobes near calyx	**44**
44	Canal of maxillary glands with heart-shaped calyx, the short proximal part with a distinct lateral protuberance and terminating with a “head of femur”-shaped dilation; foretarsal sensillum *a*’ sword-shaped	***Bolivaridia* (18)**
–	Canal of maxillary glands simple with heart-shaped calyx, more or less bipartite in the proximal part; foretarsal sensillum *a*’ shaped as an old Roman vase	***Silvestridia* (33)**
45	Abdominal appendages II–III each with three setae	**46**
–	Abdominal appendages II–III each with two setae	**52**
46	Striate band reduced	**47**
–	Striate band not reduced	**49**
47	Foretarsal sensillum *t3* knob-shaped	***Andinentulus* (14)**
–	Foretarsal sensillum *t3* not knob-shaped	**48**
48	Foretarsal sensillum *t3* relatively long, cylindrical	***Brasilidia* (20)**
–	Foretarsal sensillum *t3* willow leaf-shaped	***Fjellbergella* (41)**
49	Foretarsal sensillum *t3* very long (largely passing base of seta *α7*), cylindrical	***Najtentulus* (27)**
–	Foretarsal sensillum *t3* not cylindrical	**50**
50	Foretarsal sensillum *t3* lancet-like or finger-like and tapering on apex; canal of maxillary glands with proximal part ending with a row of small globules which increase in size and end with two globules placed beside each other	***Acerentuloides* (10)**
–	Foretarsal sensillum *t3* not lancet-like or finger-like; proximal end of canal of maxillary glands without globules	**51**
51	Foretarsal sensillum *t3* cylindrical and rounded on apex or small knob-like; sternite VIII with 4/0 setae	***Australentulus* (15)**
–	Foretarsal sensillum *t3* willow leaf-shaped; sternite VIII with 4/2 setae	***Acerentulus* (11)**
52	Canal of maxillary glands with two or three beady widenings	***Kenyentulus* (24)**
–	Canal of maxillary glands without beady widenings	**53**
53	Labial palps not reduced, with branched tuft of setae	**54**
–	Labial palps reduced, without tuft of setae (or just a one-branched terminal tuft of setae)	**61**
54	Mesonotum with three pairs of *A*-setae and metanotum with four pairs of *A*-setae; sternites with five or more *A*-setae; seta *P3* on tergites II–VI in line with other *P*-setae; striate band well developed	**55**
–	Mesonotum and metanotum with two pairs of *A*-setae; sternites with three *A*-setae; seta *P3* on tergites II–VI slightly anterior to *P*-setae row; striate band well developed or reduced	**57**
55	Foretarsal sensillum *t1* baculiform	***Yamatentomon* (47)**
–	Foretarsal sensillum *t1* claviform	**56**
56	Foretarsal sensillum *a*’ close to *t1*	***Orinentomon* (43)**
–	Foretarsal sensillum *a*’ distal to *t2* and close to *c*’	***Acerentomon* (39 – part)**
57	Striate band well developed	**58**
–	Striate band reduced	**60**
58	Foretarsal sensillum *t1* filiform; *b*’ missing	***Huashanentulus* (42)**
–	Foretarsal sensillum *t1* claviform	**59**
59	Foretarsal sensillum *t3* long parallel-sided; *b*’ present	***Maderentulus* (26)**
–	Foretarsal sensillum *t3* short lanceolate; *b*’ missing	***Sugaentulus* (44)**
60	Foretarsal sensillum *t1* claviform; *a*’ close to *t2*	***Tuxenentulus* (45)**
–	Foretarsal sensillum *t1* filiform; *a*’ close to *t1*	***Wenyingia* (46)**
61	Foretarsal sensillum *t1* baculiform	**62**
–	Foretarsal sensillum *t1* claviform	**67**
62	Foretarsal sensillum *t3* long, nearly cylindrical	***Tuxenidia* (35 – part)**
–	Foretarsal sensillum *t3* not as above	**63**
63	Head in profile has a prominent “front”	***Yinentulus* (37)**
–	Head without “front”	**64**
64	Maxillary palps with one setiform and one torch light-shaped sensillum; foretarsal sensillum *b*’ always present	***Neobaculentulus* (28)**
–	Maxillary palps with both sensilliform or setiform sensilla; foretarsal sensillum *b*’ sometimes missing	**65**
65	Canal of maxillary glands with excrescences	***Notentulus* (29)**
–	Canal of maxillary glands simple, without excrescences	**66**
66	Sternite VIII with 4/2 setae	***Yichunentulus* (48)**
–	Sternite VIII with 4/0 setae	***Baculentulus* (16)**
67	Striate band reduced	**68**
–	Striate band well developed or partly reduced (with visible parallel striae)	**73**
68	Foretarsal sensillum *b*’ present	**69**
–	Foretarsal sensillum *b*’ absent	**71**
69	Abdominal appendages II–III each with a long subapical and a very short median apical seta (< 1/3^rd^ the length of subapical one)	***Berberentulus* (17 – part)**
	Abdominal appendages II–III each with a lateral apical seta more than 1/3^rd^ the length of the subapical one	**70**
70	Labial palps reduced, with three setae and no sensilla	***Liaoxientulus* (49)**
–	Labial palps reduced with one sensillum and three setae.	***Amphientulus* (13)**
71	Foretarsal sensillum *t3* very long awl-shaped	***Amazonentulus* (12)**
–	Foretarsal sensillum *t3* not long and shaped as a willow leaf, or a small jar or as a knob	**72**
72	Proximal part of the canal of maxillary glands tripartite; abdominal appendages II–III each with the median apical seta half the length of the subapical one	***Polyadenum* (31)**
–	Canal of maxillary glands simple, with heart-shaped calyx and “single” proximal part; abdominal appendages II–III each with the median apical seta less than 1/3^rd^ the length of subapical one	***Berberentulus* (17 – part)**
73	Striate band partly reduced (the striae may be seen more or less distinctly, but never as distinct) with hook-shaped design; lateral apical seta on abdominal appendages II–III more than half as long as the subapical; sternite VIII with 4/2 setae	***Tasmanentulus* (34)**
–	Striate band well developed, without hook-shaped design (except in *Proacerella reducta* Bernard)	**74**
74	Abdominal appendages II–III each with lateral apical seta more than half as long as the subapical one; labial palps with four setae and a sensillum	***Proacerella* (32)**
–	Abdominal appendages II–III each with median apical seta less than half as long as the subapical one; labial palps with two or three setae and a sensillum	**75**
75	Labial palps with two setae and a sensillum; canal of maxillary glands with smooth and simple kidney bean-shaped calyx, the end of proximal canal slightly tripartite	***Zangentulus* (38)**
–	Labial palps with three setae and a sensillum; calyx of maxillary glands not kidney bean-shaped	**76**
76	Foretarsal sensillum *t3* small and knob-like (except in *G. sarmaticus* Shrubovych and Szeptycki where *t3* is large)	***Gracilentulus* (23)**
–	Foretarsal sensillum *t3* long, not knob-like	**77**
77	Foretarsal sensillum *b* very long reaching or passing the empodium	**78**
–	Foretarsal sensillum *b* not so long	**79**
78	Prosternum without seta *A2*	***Podolinella* (30)**
–	Prosternum with seta *A2*	***Vindobonella* (36)**
79	Canal of maxillary glands with heart-shaped calyx	***Brasilentulus* (19)**
–	Canal of maxillary glands with long and slender calyx	***Delamarentulus* (22)**
